# Modality Effectiveness in Cross-Subject Sign Language Recognition: A Skeleton Perspective

**DOI:** 10.3390/s26165184

**Published:** 2026-08-16

**Authors:** Sijie Miao, Jiaxin He, Lu Zeng

**Affiliations:** School of Computer and Software Engineering, Xihua University, Chengdu 610039, China; miaosijie@stu.xhu.edu.cn (S.M.); hejiaxin2@stu.xhu.edu.cn (J.H.)

**Keywords:** sign language recognition, cross-subject recognition, multimodal fusion, skeleton-based recognition, modality effectiveness

## Abstract

Accessibility-oriented sign language recognition plays an important role in intelligent human–computer interaction, but cross-subject recognition remains challenging because test signers are unseen during training. Variations in appearance, motion style, and modality quality among different signers introduce significant distribution differences between training and testing samples, limiting model generalization. Although multimodal approaches commonly assume that RGB, skeleton, and depth modalities provide complementary information, their effectiveness under cross-subject recognition remains insufficiently explored. This study investigates modality effectiveness in SLR500 word-level sign language recognition under a cross-subject protocol. A unified evaluation framework is established to compare RGB-only, skeleton-only, RGB–skeleton fusion, and RGB–skeleton–depth fusion under consistent data splits, preprocessing procedures, training settings, and evaluation metrics. Sample-level prediction transitions are further analyzed to examine how fusion changes recognition outcomes relative to the skeleton-based reference model. The results show that the skeleton modality provides more reliable recognition performance under the evaluated cross-subject setting, while the tested fusion strategies do not consistently improve over the skeleton-only configuration. Based on the modality-effectiveness analysis, a skeleton-based reference model is established using a Spatial–Temporal Graph Convolutional Network (ST-GCN)-based spatio-temporal encoder, learned confidence-weighted temporal pooling, and a lightweight classifier. Multi-seed experiments further verify the stability of the model, which achieves 86.37% Top-1 accuracy and 96.86% Top-5 accuracy on the SLR500 cross-subject test set. These results emphasize the importance of evaluating modality reliability before designing complex fusion strategies for cross-subject sign language recognition.

## 1. Introduction

With the rapid development of artificial intelligence, computer vision, and human–computer interaction, sign language recognition (SLR) has become an important research direction for accessible intelligent interaction. As a major communication approach for deaf and hard-of-hearing communities, SLR has attracted increasing attention due to its potential applications in intelligent services, human–computer interaction, and inclusive communication systems. Recent advances in computer vision and deep learning have promoted the transition of SLR from early sensor-based approaches toward non-intrusive visual recognition based on RGB videos, depth information, and human pose representations [1]. However, reliable recognition remains challenging under cross-subject settings, where test signers are unseen during training. Variations in appearance, motion style, body structure, and acquisition conditions introduce distribution shifts between training and testing samples, making modality reliability and generalization capability critical issues.

SLR contains several related but distinct tasks, including isolated sign language recognition (ISLR), continuous sign language recognition (CSLR), and sign language translation [2]. ISLR aims to recognize individual isolated signs from predefined categories and output corresponding class labels, which is mainly applied in vocabulary-level sign recognition and intelligent human–computer interaction. CSLR focuses on recognizing continuous sign sequences from unsegmented videos and requires temporal modeling to predict ordered gloss sequences. Sign language translation further extends CSLR by converting continuous sign expressions into natural language sentences, requiring additional semantic understanding and language generation capabilities for applications such as cross-modal communication and automatic translation systems. For example, Sign Language Transformers jointly address continuous sign language recognition and translation through Transformer-based sequence modeling and Connectionist Temporal Classification (CTC) optimization [3]. Compared with CSLR and translation, ISLR provides a more controlled setting for investigating visual representation reliability because each sample corresponds to a predefined sign category. Therefore, this study focuses on cross-subject ISLR, where unseen signer variations introduce distribution differences and make modality effectiveness an important factor for recognition robustness.

The development of large-scale ISLR datasets has further promoted research toward realistic recognition scenarios involving larger vocabularies and diverse signers. WLASL provides a large-scale word-level American Sign Language benchmark with extensive sign categories and signer variations [4]. MS-ASL further introduces real-world ASL videos with large vocabulary sizes and signer-independent evaluation protocols [5]. AUTSL provides a multimodal Turkish Sign Language dataset containing RGB, depth, and skeleton modalities, enabling the evaluation of multimodal recognition methods under user-independent settings [6]. These datasets demonstrate that modern ISLR systems should not only achieve high recognition accuracy but also maintain robustness across unseen signers.

RGB-based methods directly learn visual representations from video sequences by exploiting appearance and motion information. Two-stream networks and 3D convolutional networks demonstrate the effectiveness of jointly modeling spatial and temporal information for video understanding [7,8]. Subsequent models, including Inception-based architectures, I3D, 3D ResNet, Temporal Segment Networks (TSNs), and SlowFast networks, further improve visual feature extraction and temporal representation learning [9,10,11,12,13]. Video-language representation learning further investigates joint visual and semantic modeling through approaches such as VideoBERT [14]. Meanwhile, non-local operations and Transformer-based video models improve long-range dependency modeling in visual sequences [15,16,17,18]. However, RGB representations remain sensitive to illumination, background, viewpoint, and signer-specific appearance variations, which may limit generalization under cross-subject ISLR.

Skeleton-based methods represent human motion using structured keypoint sequences and reduce dependence on appearance-related factors. Early graph-based representation learning methods, including spectral graph convolution and graph attention networks, provide important foundations for topology-aware modeling [19,20,21]. ST-GCN further extends graph convolution to skeleton-based action recognition by modeling human joints as spatial–temporal graphs [22]. Subsequent methods improve skeleton modeling through adaptive graph construction and topology refinement strategies [23,24,25]. In sign language recognition, SAM-SLR, SignBERT, and skeleton Transformer-based methods demonstrate the effectiveness of structural representations for capturing motion patterns and long-range dependencies [26,27,28]. Nevertheless, skeleton representations may lose fine-grained appearance information, such as hand shape and facial expression, and their effectiveness depends on pose estimation quality. Therefore, the relative effectiveness of skeleton representations compared with RGB and depth modalities remains an open question under cross-subject ISLR.

Multimodal fusion has been widely investigated in sign language recognition because different modalities may provide complementary information. RGB captures appearance-related cues, skeleton describes body topology and motion dynamics, and depth provides additional spatial information. Existing studies explore multi-cue modeling, alignment strategies, and cross-modal augmentation to integrate heterogeneous information for sign language recognition [26,29,30,31,32]. However, effective multimodal learning depends not only on the number of modalities but also on representation compatibility, modality reliability, and interaction quality. General multimodal learning studies emphasize the importance of cross-modal representation learning and modality interaction mechanisms [33,34]. Recent multimodal vision studies further investigate adaptive modality interaction and information selection mechanisms, such as dual-modality interaction learning and prior-guided state-space modeling [35,36]. In addition, transfer learning studies indicate that auxiliary information with insufficient relevance may fail to improve the target task and can introduce negative effects [37,38]. Therefore, before designing increasingly complex fusion architectures, it is necessary to systematically evaluate whether different modalities provide reliable and complementary information under a unified cross-subject protocol.

Although existing studies have achieved significant progress in RGB modeling, skeleton representation learning, and multimodal fusion, several issues remain insufficiently explored. First, most existing methods focus on designing stronger architectures, while the relative effectiveness of different modalities under identical evaluation protocols is less systematically investigated. Second, whether auxiliary modalities consistently improve recognition performance when their quality differs across unseen signers remains unclear. Third, overall accuracy alone cannot reveal how fusion changes individual prediction outcomes compared with a single-modality reference model.

To address these issues, this study conducts a controlled empirical analysis of modality effectiveness for SLR500 word-level sign language recognition under a cross-subject setting. RGB-only, skeleton-only, RGB–skeleton fusion, and RGB–skeleton–depth fusion configurations are evaluated under consistent data splits, preprocessing procedures, and evaluation metrics. Instead of assuming that increasing the number of modalities always improves performance, this study investigates modality reliability and fusion behavior through controlled comparisons and sample-level prediction transition analysis. Based on the observed modality effectiveness, a skeleton-based reference model is established using ST-GCN-based spatio-temporal feature extraction, learned confidence-weighted temporal pooling, and a lightweight classifier.

The main contributions of this study are summarized as follows:

1. A controlled modality-effectiveness evaluation framework is established for cross-subject word-level sign language recognition. RGB, skeleton, depth, and fusion configurations are systematically compared to analyze the reliability of different visual modalities under a unified protocol.
2. A sample-level prediction transition analysis is introduced to investigate how multimodal fusion changes recognition outcomes relative to a skeleton-based reference model, providing a more detailed understanding beyond overall accuracy.
3. A skeleton-based reference model is developed based on the modality-effectiveness analysis. By integrating ST-GCN-based spatio-temporal representation learning, learned confidence-weighted temporal pooling, and a lightweight classifier, the model provides an effective solution for cross-subject ISLR.

The remainder of this paper is organized as follows. Section 2 introduces the problem formulation, dataset protocol, preprocessing procedure, modality-effectiveness framework, skeleton-based reference model, and implementation details. Section 3 presents experimental results, including modality-effectiveness evaluation, overall performance, external validation, same-protocol baseline comparison, ablation studies, sequence-length sensitivity analysis, stability analysis, statistical analysis, convergence analysis, error analysis, and fusion behavior analysis. Section 4 discusses modality reliability, fusion effectiveness, and limitations. Section 5 concludes the paper and outlines future work.

## 2. Materials and Methods

### 2.1. Problem Formulation and Modality-Effectiveness Objective

Cross-subject isolated sign language recognition (ISLR) aims to classify isolated sign samples from signers that are not observed during training. Let $S_{train}$, $S_{val}$, and $S_{test}$ denote the signer sets used for training, validation, and testing, respectively. Under the cross-subject protocol, these signer sets are mutually disjoint:

(1) $$S_{train}\cap S_{val}=⌀,\;S_{train}\cap S_{test}=⌀,\;S_{val}\cap S_{test}=⌀.$$

Given a sign sample $X_{i}$ with class label $y_{i}\in \left\{1,\ldots ,K\right\}$, where K=500, the objective of ISLR is to learn a recognition function:

(2) $${\hat{y}}_{i}=f_{\theta }\left(X_{i}\right),$$

where $f_{\theta }$ denotes the recognition model.

To evaluate modality effectiveness, each sign sample is represented by available visual modalities, including RGB, skeleton, and depth information:

(3) $$X_{i}=\left\{X_{i}^{rgb},X_{i}^{sk},X_{i}^{depth}\right\},$$

where $X_{i}^{rgb}$, $X_{i}^{sk}$, and $X_{i}^{depth}$ denote the RGB, skeleton, and depth modalities, respectively. The available modality set is defined as

(4) M={rgb,sk,depth}.

For a selected modality configuration C⊆M, the corresponding input is denoted as $X_{i}^{C}$, and the prediction is formulated as

(5) $${\hat{y}}_{i}^{C}=f_{\theta }^{C}\left(X_{i}^{C}\right).$$

The objective of this study is not to design increasingly complex fusion architectures but to empirically investigate the reliability and contribution of different modality configurations under a unified cross-subject protocol. Therefore, RGB-only, skeleton-only, RGB–skeleton fusion, and RGB–skeleton–depth fusion configurations are systematically compared to analyze their effectiveness and interaction behavior.

For a modality configuration C, the Top-1 accuracy on the test set is defined as

(6) $$A^{C}=\frac{1}{|D_{test}|}\sum_{\left(X_{i},y_{i}\right)\in D_{test}}I\left({\hat{y}}_{i}^{C}=y_{i}\right),$$

where $D_{test}$ denotes the test set and I(·) is the indicator function. This formulation is used only for performance reporting and comparison, rather than test-set-based model selection or optimization.

In addition to overall accuracy, this study further analyzes sample-level prediction transitions between the skeleton-based reference model and multimodal fusion models. Given the prediction ${\hat{y}}_{i}^{sk}$ from the skeleton-based reference model and the prediction ${\hat{y}}_{i}^{fusion}$ from an evaluated fusion configuration, two prediction transition categories are defined:

(7) $$T_{degraded}=\left\{i|{\hat{y}}_{i}^{sk}=y_{i},\;{\hat{y}}_{i}^{fusion}\neq y_{i}\right\},$$

(8) $$T_{corrected}=\left\{i|{\hat{y}}_{i}^{sk}\neq y_{i},\;{\hat{y}}_{i}^{fusion}=y_{i}\right\}.$$

Here, $T_{degraded}$ represents samples that are correctly recognized by the skeleton-based reference model but incorrectly classified after fusion, while $T_{corrected}$ represents samples that are successfully corrected by fusion.

To quantitatively measure fusion-induced prediction changes, two transition ratios are further defined:

(9) $$R_{degraded}=\frac{|T_{degraded}|}{|D_{test}|},$$

(10) $$R_{corrected}=\frac{|T_{corrected}|}{|D_{test}|}.$$

The difference between these two ratios reflects whether the evaluated fusion strategy is associated with more prediction degradation than correction under the cross-subject setting.

### 2.2. Dataset and Cross-Subject Protocol

The primary experiments in this study are conducted on the SLR500 word-level sign language dataset. SLR500 contains 500 sign categories collected from multiple signers, providing a suitable benchmark for evaluating isolated sign language recognition under cross-subject settings. Following the formulation in Section 2.1, the training, validation, and test sets are constructed using non-overlapping signer identities. Specifically, the adopted split contains 95,146 training samples, 12,166 validation samples, and 12,162 test samples. This protocol ensures that test samples are performed by unseen signers, enabling the evaluation of modality reliability and recognition generalization under inter-subject variations.

To ensure a fair modality-effectiveness evaluation, a unified sample index is constructed before training and evaluation. RGB sequences, skeleton sequences, and depth sequences are aligned at the sample level, and only samples with complete modality records and sufficient valid frames are retained. Based on this unified protocol, RGB-only, skeleton-only, RGB–skeleton fusion, RGB–skeleton–depth fusion, and different fusion strategies are evaluated under the same cross-subject split and evaluation settings. In the final skeleton-based reference model, only the skeleton modality is enabled, while RGB, depth, and bounding-box inputs are disabled. This design allows the contribution of skeleton representations to be independently examined under the same recognition protocol.

In addition to the primary SLR500 experiments, AUTSL is used as an external validation dataset to evaluate the transferability of the skeleton-based reference model. AUTSL is a large-scale multimodal Turkish Sign Language dataset containing 226 sign categories performed by 43 signers, with RGB, depth, and skeleton modalities recorded for each sample. The dataset provides a user-independent evaluation setting, making it suitable for assessing recognition performance on unseen signers. In this study, AUTSL is not used for modality-effectiveness comparison or model selection; instead, it is only used to evaluate whether the skeleton-based reference model maintains stable recognition performance on an additional user-independent dataset.

### 2.3. Data Preprocessing

The preprocessing pipeline is designed to support both the modality-effectiveness evaluation and the final skeleton-based reference model. For RGB-related configurations, RGB videos are decoded into frame sequences and processed using the same frame sampling strategy within each experimental setting. All RGB-related configurations follow the same preprocessing protocol to ensure a fair comparison across modality settings. The number of sampled frames follows the corresponding RGB experiment configuration so that RGB-only and RGB-related fusion models are evaluated under consistent input settings. For depth-related configurations, depth sequences are aligned with the corresponding sign samples and used as an auxiliary modality in the modality-effectiveness evaluation. No additional depth-specific enhancement is introduced. Since the final skeleton-based reference model uses skeleton sequences as the primary input, the skeleton preprocessing procedure is described in detail below.

For each skeleton sample, the original joint sequence is represented as

(11) $$X^{sk}=\left\{\left(q_{t,v},c_{t,v}\right)|t=1,\ldots ,T,\;v=1,\ldots ,V\right\},$$

where *T* denotes the original number of frames and V=25 denotes the number of body joints extracted from each frame. Each joint contains a two-dimensional coordinate vector and a confidence score:

(12) $$q_{t,v}=\left(x_{t,v},y_{t,v}\right),\;c_{t,v}\in \left[0,1\right],$$

where $q_{t,v}$ denotes the coordinate of the *v*-th joint at the *t*-th frame, and $c_{t,v}$ denotes its confidence score.

To reduce the influence of body-scale and position differences among different signers, spatial normalization is applied to the skeleton coordinates. Let $q_{t,ref}$ denote the reference center joint and $d_{t}$ denote the scale factor. The normalized joint coordinate is defined as

(13) $${\tilde{q}}_{t,v}=\frac{q_{t,v}-q_{t,ref}}{d_{t}}.$$

The scale factor is computed from the distance between predefined upper-body reference joints. This normalization reduces individual scale differences and encourages the model to focus on relative joint structures rather than absolute body position and scale.

For low-confidence joints, a combination of missing-value marking and local linear interpolation is used. When the confidence score $c_{t,v}$ of a joint is lower than a predefined threshold, the joint is marked as missing. If the missing interval is short, the joint coordinate is interpolated according to the adjacent valid frames:

(14) $$q_{t,v}=q_{t_{1},v}+\frac{t-t_{1}}{t_{2}-t_{1}}\left(q_{t_{2},v}-q_{t_{1},v}\right),$$

where $t_{1}$ and $t_{2}$ denote the valid frames before and after the missing interval, respectively. For long continuous missing intervals, forced interpolation is not applied, in order to avoid introducing unreliable motion trajectories.

In addition, bone vectors and joint angles are computed to enrich the skeleton representation. For two connected joints *i* and *j* at frame *t*, the bone vector is defined using normalized coordinates:

(15) $$b_{t,i,j}={\tilde{q}}_{t,j}-{\tilde{q}}_{t,i}.$$

For two adjacent bone vectors $b_{t,i,j}$ and $b_{t,j,k}$, the joint angle is computed as

(16) $$\alpha_{t,i,j,k}=\arccos\left(\frac{b_{t,i,j}\cdot b_{t,j,k}}{{∥b_{t,i,j}∥}_{2}{∥b_{t,j,k}∥}_{2}+\epsilon }\right),$$

where ϵ is a small constant used to avoid division by zero.

After spatial normalization, missing-joint handling, and feature construction, each skeleton sample is represented as a variable-length feature sequence. To obtain a fixed-length input, all skeleton sequences are temporally aligned to $T_{s}$ frames. Sequences shorter than $T_{s}$ frames are expanded by interpolation, whereas sequences longer than $T_{s}$ frames are compressed by uniform sampling. After temporal alignment, each skeleton sample is represented as a fixed-size feature tensor:

(17) $$X^{\prime }\in R^{T_{s}\times V\times C_{s}},$$

where $T_{s}$ denotes the fixed temporal length after resampling, *V* denotes the number of skeleton joints, and $C_{s}$ denotes the constructed feature dimension. In this study, $T_{s}=64$, V=25, and $C_{s}=75$. Therefore, $X^{\prime }$ represents the processed feature tensor of a single skeleton sample. The influence of different temporal lengths is further investigated through a sequence-length sensitivity analysis in Section 3.5.

Meanwhile, the raw frame-level confidence score is calculated by averaging the confidence scores of all joints in a frame:

(18) $${\bar{c}}_{t}=\frac{1}{V}\sum_{v=1}^{V}c_{t,v}.$$

The final skeleton-based model input consists of the skeleton feature tensor $X^{\prime }$ and the corresponding raw frame-level confidence sequence $\bar{c}={\left\{{\bar{c}}_{t}\right\}}_{t=1}^{T_{s}}$. The raw confidence sequence is not directly used as temporal weights; instead, it is transformed through a learnable confidence encoder to generate adaptive temporal weighting information in the skeleton-based reference model.

### 2.4. Modality-Effectiveness Evaluation Framework

This subsection describes the controlled modality-effectiveness evaluation framework used to compare different modality configurations before establishing the skeleton-based reference model. The objective of this framework is not to design a new multimodal model but to provide a unified experimental protocol for analyzing modality reliability and fusion behavior under the cross-subject ISLR setting.

Word-level sign language recognition involves multiple visual cues, including hand appearance, facial expression, body posture, joint motion, and spatial structure. Different modalities capture different aspects of these cues. RGB videos provide rich appearance information but may be sensitive to background, illumination, clothing, viewpoint, and signer-specific appearance variations. Skeleton sequences describe body topology and motion dynamics through keypoint trajectories while reducing dependence on appearance-related factors. However, skeleton representations may lose fine-grained visual information, such as detailed hand shape and facial expression. Depth information provides additional spatial structure, although its effectiveness depends on data quality and temporal alignment.

Based on the unified sample index described in Section 2.2, RGB, skeleton, and depth inputs are aligned at the sample level. RGB-only, skeleton-only, RGB–skeleton fusion, RGB–skeleton–depth fusion, and different fusion strategies are evaluated under the same data split, preprocessing pipeline, training strategy, and evaluation metrics. This controlled setting enables a fair comparison of modality contribution and fusion behavior under the cross-subject protocol.

**Figure 1:**
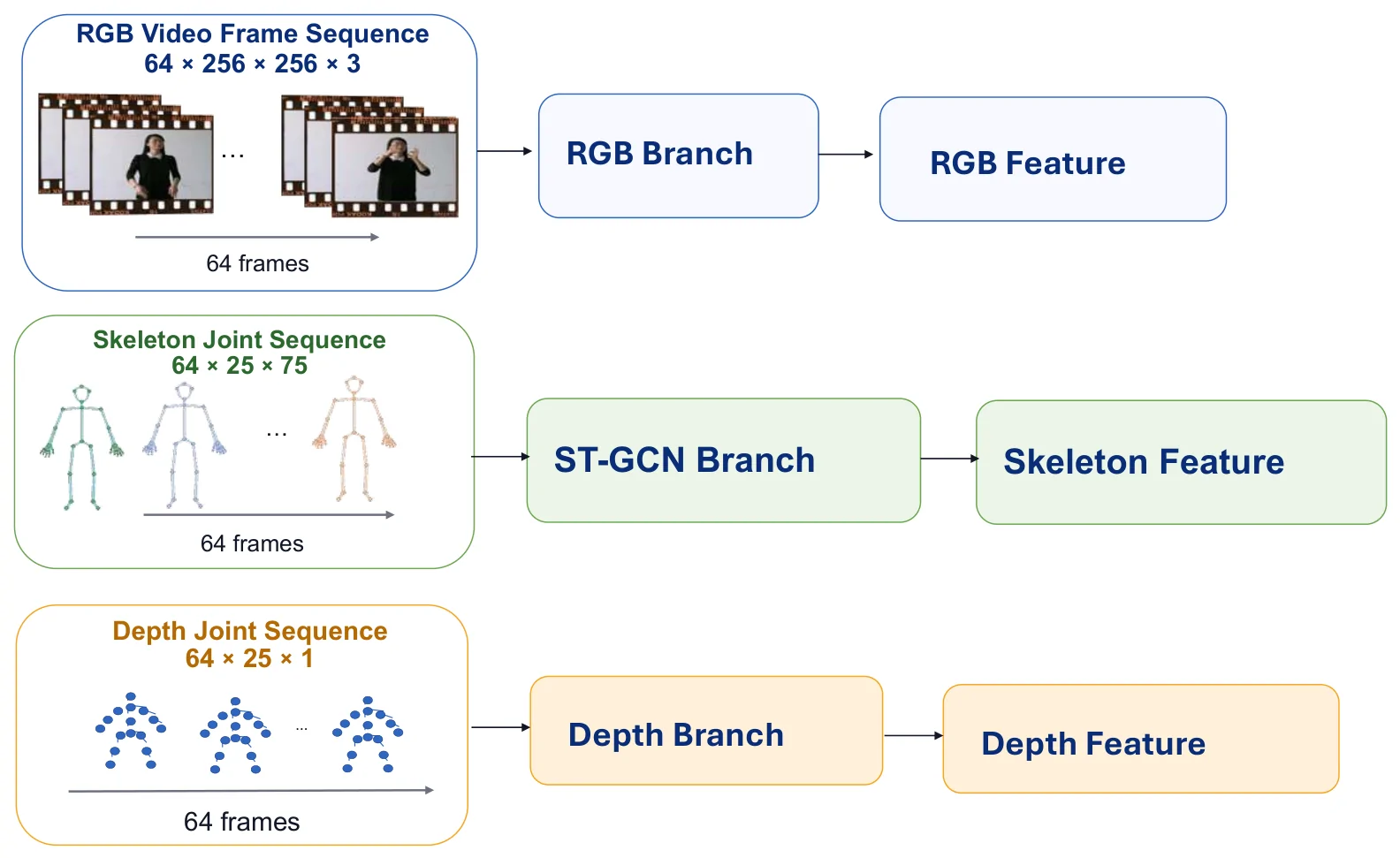
Overview of modality-specific processing branches used for controlled modality-effectiveness evaluation. RGB, skeleton, and depth modalities are independently processed to obtain modality-specific representations under the unified cross-subject protocol.

The modality-specific processing branches used for controlled modality-effectiveness evaluation are illustrated in Figure 1. For a selected modality configuration C⊆{rgb,sk,depth}, the feature representation of modality m∈C is formulated as

(19) $$F^{\left(m\right)}=\phi_{m}\left(X^{\left(m\right)}\right),$$

where $X^{\left(m\right)}$ denotes the input sequence of modality *m* and $\phi_{m}\left(\cdot \right)$ represents the corresponding modality-specific feature extractor.

For single-modality configurations, the extracted representation is directly used for recognition. For multimodal configurations, different fusion strategies are evaluated to investigate whether additional modalities provide complementary information under the cross-subject setting:

(20) $$Z=\Psi_{C}\left(\{F^{\left(m\right)}|m\in C\}\right),$$

where $\Psi_{C}\left(\cdot \right)$ denotes the fusion operation of the evaluated modality configuration. In this study, simple RGB–skeleton fusion, MAGF fusion strategy, residual RGB–skeleton fusion, and RGB–skeleton–depth fusion are evaluated as representative configurations.

The fused or single-modality representation is subsequently fed into the recognition module:

(21) $$\hat{y}=g_{C}\left(Z\right),$$

where $g_{C}\left(\cdot \right)$ denotes the recognition module associated with the corresponding configuration. All configurations are evaluated using Top-1 and Top-5 accuracy under the same cross-subject test protocol.

**Figure 2:**
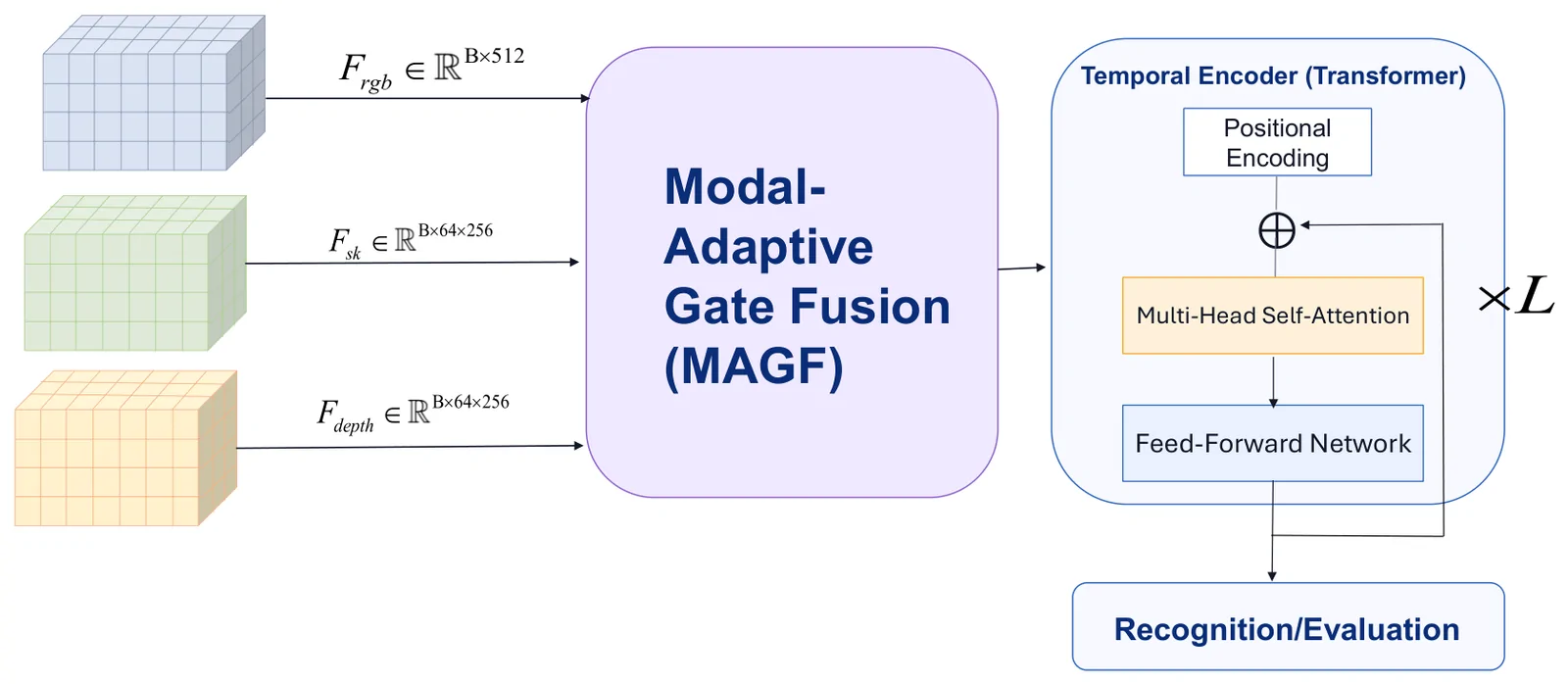
Overview of the fusion evaluation pipeline. Different modality representations are combined through multiple fusion strategies and evaluated under the same recognition protocol.

The fusion evaluation pipeline is illustrated in Figure 2. Different modality representations are combined through various fusion strategies and evaluated using the corresponding recognition module.

This framework provides a controlled basis for comparing modality contribution and fusion behavior. By maintaining consistent data splits, sample indices, preprocessing procedures, training settings, and evaluation metrics, the influence of uncontrolled experimental factors is reduced. Therefore, the observed performance differences are analyzed to understand modality effectiveness and fusion-induced performance changes, which further motivate the establishment of the skeleton-based reference model.

### 2.5. Skeleton-Based Reference Model

Based on the modality-effectiveness evaluation, a skeleton-based reference model is established to examine whether structural motion representations can provide reliable recognition performance under the cross-subject setting. Rather than assuming that additional auxiliary modalities always provide complementary information, this model focuses on exploiting skeleton-based structural information and provides a stable reference for analyzing modality effectiveness.

**Figure 3:**
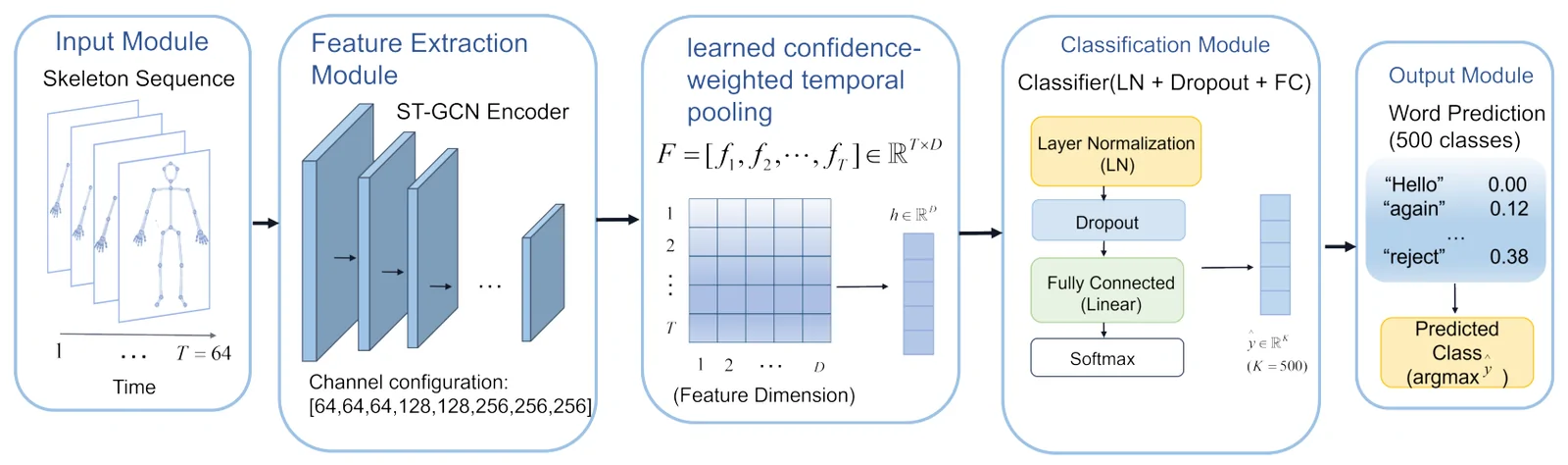
Overall architecture of the skeleton-based reference model. The model consists of an ST-GCN encoder, learned confidence-weighted temporal pooling, and a lightweight LayerNorm–Dropout–Linear classification head.

ST-GCN is adopted as the spatio-temporal feature extractor because it can model spatial joint dependencies and temporal motion patterns in skeleton sequences [22]. The skeleton-based reference model performs word-level sign classification through ST-GCN-based feature extraction, learned confidence-weighted temporal pooling, and a lightweight LayerNorm–Dropout–Linear classification head. The overall architecture is illustrated in Figure 3.

The overall model is formulated as

(22) $$\hat{y}=f_{cls}\left(f_{cwpool}\left(f_{ST-GCN}\left(X^{\prime }\right),\bar{c}\right)\right),$$

where $X^{\prime }\in R^{T_{s}\times V\times C_{s}}$ denotes the preprocessed skeleton feature tensor, and $\bar{c}$ denotes the raw frame-level confidence sequence. $f_{ST-GCN}$ represents the spatio-temporal graph convolutional encoder, $f_{cwpool}$ denotes the learned confidence-weighted temporal pooling module, and $f_{cls}$ represents the lightweight classification head. In this study, $T_{s}=64$, V=25, and $C_{s}=75$.

**Figure 4:**
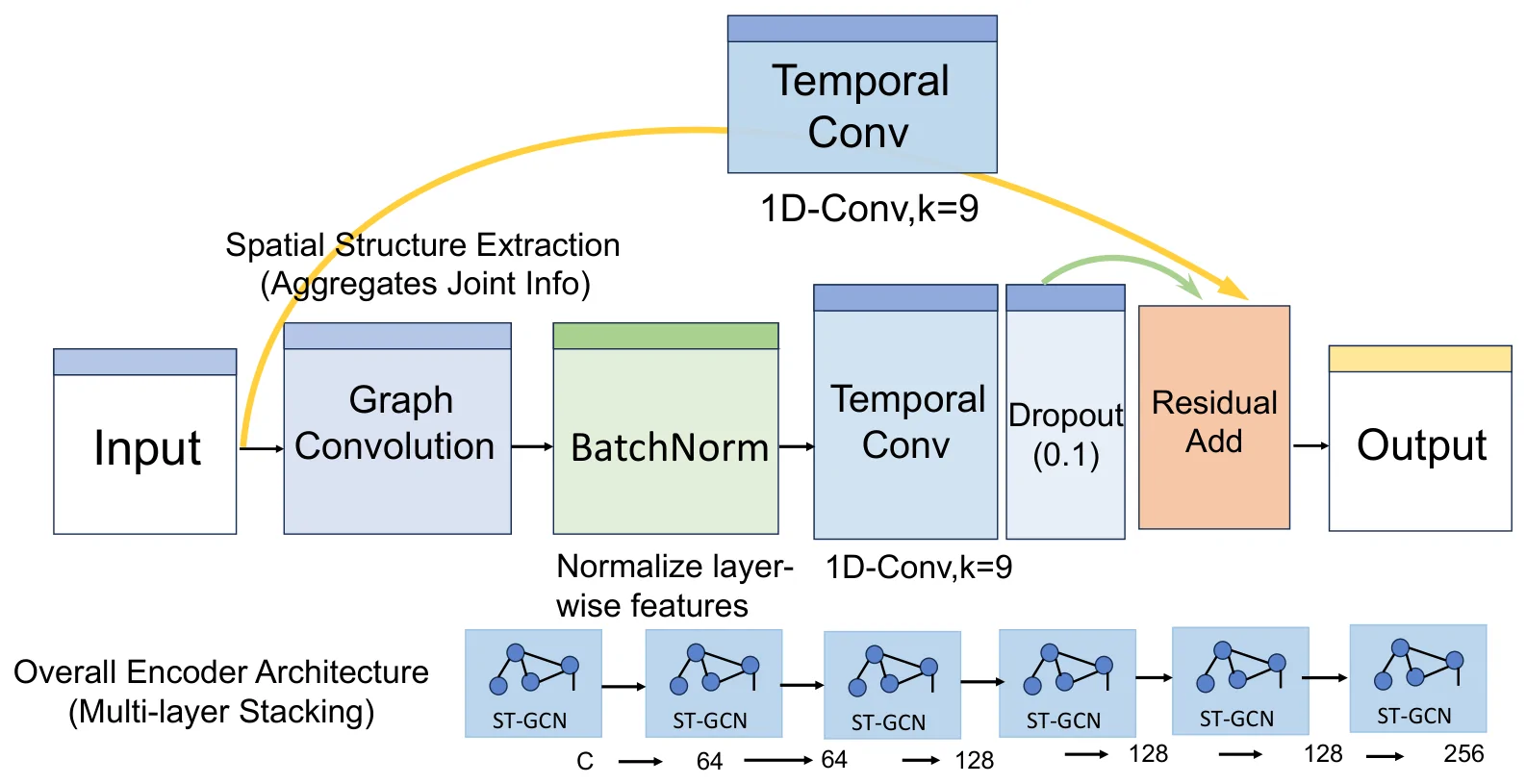
Structure of the ST-GCN encoder used for skeleton feature extraction.

To extract spatio-temporal representations from skeleton sequences, the skeleton input is represented as a graph:

(23) G=(V,E),

where V represents the skeleton joints and E denotes the natural body connections between joints. The structure of the ST-GCN encoder is illustrated in Figure 4.

The spatial graph convolution operation is formulated as

(24) $$H^{\left(l+1\right)}=\sigma \left(\tilde{A}H^{\left(l\right)}W^{\left(l\right)}\right),$$

where $\tilde{A}$ denotes the normalized adjacency matrix, $H^{\left(l\right)}\in R^{V\times D}$ represents the feature matrix of the *l*-th layer, $W^{\left(l\right)}\in R^{D\times D^{\prime }}$ is the learnable transformation matrix, and σ(·) denotes the nonlinear activation function.

In the temporal dimension, one-dimensional convolution is applied to model motion dynamics across consecutive frames:

(25) $$H^{\prime }=\mathrm{Conv}1D\left(H\right),$$

where *H* denotes the input skeleton feature sequence and $H^{\prime }$ represents the temporally enhanced feature representation.

The ST-GCN encoder consists of multiple spatio-temporal graph convolution units with the following channel configuration:

(26) [64,64,64,128,128,128,256,256,256].

Each unit contains spatial graph convolution, temporal convolution, residual connection, batch normalization, and dropout operations [22,39,40,41]. This structure enables the model to capture both spatial topology and temporal motion patterns of skeleton sequences.

After ST-GCN encoding, the output feature sequence is represented as

(27) $$F=\left[F_{1},F_{2},\ldots ,F_{T_{s}}\right],\;F_{t}\in R^{D}.$$

To obtain a fixed-length representation, a learned confidence-weighted temporal pooling module is applied. The raw frame-level confidence score ${\bar{c}}_{t}$ is first transformed through a confidence encoder:

(28) $${\hat{c}}_{t}=\sigma \left(W_{2}\mathrm{ReLU}\left(W_{1}{\bar{c}}_{t}+b_{1}\right)+b_{2}\right),$$

where the confidence encoder follows a Linear(1,16)–ReLU–Linear(16,1)–Sigmoid structure.

The confidence scores are normalized along the temporal dimension:

(29) $$w_{t}=\frac{{\hat{c}}_{t}}{\sum_{k=1}^{T_{s}}{\hat{c}}_{k}+10^{-6}}.$$

The final sequence-level representation is obtained through weighted temporal pooling:

(30) $$h=\sum_{t=1}^{T_{s}}w_{t}F_{t}.$$

This design enables the model to learn the contribution of different frames according to confidence information, instead of directly applying raw confidence scores as fixed temporal weights.

For classification, a lightweight LayerNorm–Dropout–Linear classification head is adopted:

(31) $$\hat{y}=\mathrm{Softmax}\left(W\cdot \mathrm{Dropout}\left(\mathrm{LN}\left(h\right)\right)+b\right),$$

where *W* and *b* denote the learnable parameters of the classifier.

It should be emphasized that the skeleton-based model is not designed as a novel graph neural network architecture. Instead, it serves as a reference model established from the modality-effectiveness analysis. By focusing on a structurally informative modality and avoiding additional complex fusion components, this model provides a clear basis for evaluating skeleton representation effectiveness under cross-subject ISLR settings.

### 2.6. Training Strategy and Evaluation Metrics

#### 2.6.1. Implementation Details

All experiments follow the same cross-subject evaluation protocol. The skeleton-based reference model is implemented using PyTorch. The input skeleton sequence is uniformly resampled to 64 frames. After feature construction, each joint is represented by a 75-dimensional feature vector, including normalized coordinates, bone vectors, and joint-angle information.

The backbone network adopts a nine-block ST-GCN architecture with a temporal kernel size of 9. The channel configuration of the ST-GCN encoder is:

(32) [64,64,64,128,128,128,256,256,256].

After spatio-temporal feature extraction, joint-level features are aggregated into frame-level representations. The raw frame-level skeleton confidence scores are processed through a two-layer confidence encoder consisting of Linear(1,16), ReLU, Linear(16,1), and Sigmoid operations. The encoded confidence scores are normalized along the temporal dimension and used for learned confidence-weighted temporal pooling, producing a compact sequence-level representation for classification.

The model is optimized using AdamW with an initial learning rate of $1\times 10^{-4}$ and weight decay of $5\times 10^{-4}$. A cosine annealing scheduler is adopted during training. Cross-entropy loss is used as the optimization objective, and gradient clipping with a global norm of 1.0 is applied to improve optimization stability.

To evaluate training robustness, three independent runs with different random seeds (42, 123, and 3407) are conducted while keeping the dataset split, preprocessing procedure, and training configuration unchanged. For each run, the checkpoint with the highest validation Top-1 accuracy is selected. The multi-seed results are reported in the stability analysis section using mean ± standard deviation.

The software environment consists of Windows 11, Python 3.9.25, PyTorch 2.5.1, and CUDA 11.8. The experiments are conducted on a workstation equipped with an Intel Core i7-13620H processor, 16 GB system memory, and an NVIDIA RTX 4090 Laptop GPU.

#### 2.6.2. Computational Cost

To provide additional information regarding computational efficiency and reproducibility, the parameter number, computational complexity, and peak training memory of the skeleton-based reference model are summarized in Table 1. The computational cost is measured under the default 64-frame skeleton input setting.

#### 2.6.3. Optimization and Evaluation Metrics

The skeleton-based reference model is optimized using the cross-entropy loss:

(33) $$L=-\frac{1}{N}\sum_{n=1}^{N}\sum_{k=1}^{K}y_{n,k}\log\left({\hat{p}}_{n,k}\right),$$

where *N* denotes the number of samples in a mini-batch, K=500 represents the number of sign categories, $y_{n,k}$ denotes the one-hot ground-truth label of the *n*-th sample for class *k*, and ${\hat{p}}_{n,k}$ denotes the predicted probability of class *k*. The skeleton-based reference model is optimized only with the classification objective and does not introduce auxiliary detection losses, confidence prediction losses, or additional multi-task objectives, which keeps the optimization target explicit.

Top-1 accuracy and Top-5 accuracy are adopted as the primary evaluation metrics. Top-1 accuracy measures whether the highest-ranked prediction matches the ground-truth label, while Top-5 accuracy evaluates whether the correct category is included among the five highest-ranked predictions. For r∈{1,5}, Top-*r* accuracy is defined as

(34) $$Acc@r=\frac{1}{\left|D\right|}\sum_{(X_{n},y_{n})\in D}I\left(y_{n}\in {\mathrm{Top}}_{r}\left({\hat{p}}_{n}\right)\right),$$

where D denotes the evaluation set, ${\hat{p}}_{n}$ denotes the predicted probability distribution of the *n*-th sample, ${\mathrm{Top}}_{r}\left({\hat{p}}_{n}\right)$ denotes the set of the *r* highest-ranked predicted classes, and I(·) is the indicator function.

For error analysis, the error rate is calculated as

(35) E=1−A,

where *A* denotes the corresponding accuracy and *E* represents the error rate. The error rate is further used to analyze fusion-induced performance changes under different modality configurations. The detailed stability results based on different random seeds are reported in Section 3.6.

## 3. Results

This section presents the experimental results of the modality-effectiveness evaluation and the skeleton-based reference model. We first compare RGB-only, skeleton-only, RGB–skeleton fusion, and RGB–skeleton–depth fusion under the unified cross-subject evaluation framework to examine the relative reliability of different modality configurations. Based on the observed modality behavior, we then report the performance of the skeleton-based reference model on SLR500 and provide external validation on AUTSL. A reproduced representative pose/skeleton-based baseline is further evaluated under the same SLR500 cross-subject protocol to provide a direct comparison with the skeleton-based reference model. Finally, ablation studies, sequence-length sensitivity analysis, stability analysis, statistical significance analysis, convergence analysis, error-pattern analysis, and sample-level prediction transition analysis are provided to further explain the effectiveness of skeleton representations and the fusion-induced performance changes observed under the current setting.

### 3.1. Modality-Effectiveness Evaluation Results

Under the unified modality-effectiveness evaluation framework, RGB-only, skeleton-only, RGB–skeleton fusion, and RGB–skeleton–depth fusion configurations are evaluated. All configurations are compared under the same cross-subject data split and evaluation metrics, while branch-specific input settings are kept consistent within each corresponding experiment. The purpose of this evaluation is to investigate the relative effectiveness and reliability of different modality configurations rather than to report the final optimized skeleton-based recognition performance. The results are shown in Table 2.

As shown in Table 2, different modality configurations exhibit substantially different recognition performance under the cross-subject setting. The RGB-only configurations achieve 2.6% and 3.0% Top-1 accuracy with three-frame and eight-frame inputs, respectively. In contrast, the skeleton-only configuration achieves 55.3% accuracy, indicating that skeleton representations provide more reliable structural information than the evaluated RGB configurations under the current setting.

The multimodal results further show that the evaluated fusion strategies do not consistently improve recognition performance over the skeleton-only configuration. The simple RGB–skeleton fusion and MAGF-based fusion both achieve 54.0% accuracy, while residual RGB–skeleton fusion achieves results ranging from 51.3% to 54.0%. The RGB–skeleton–depth fusion configuration achieves 2.24% accuracy, indicating that the additional depth modality does not provide effective complementary information under the evaluated setting. These results suggest that the effectiveness of multimodal fusion depends not only on the number of available modalities, but also on the reliability and compatibility of modality representations.

The influence of RGB temporal sampling is further investigated. For RGB-only configurations, increasing the number of sampled frames from three to eight frames provides only a limited improvement from 2.6% to 3.0%. Meanwhile, in residual RGB–skeleton fusion, increasing RGB frames from 3 to 16 decreases the accuracy from 53.3% to 51.3%. These results indicate that simply increasing temporal input length does not guarantee improved recognition performance when the additional visual information does not provide sufficiently discriminative representations under the cross-subject setting.

**Figure 5:**
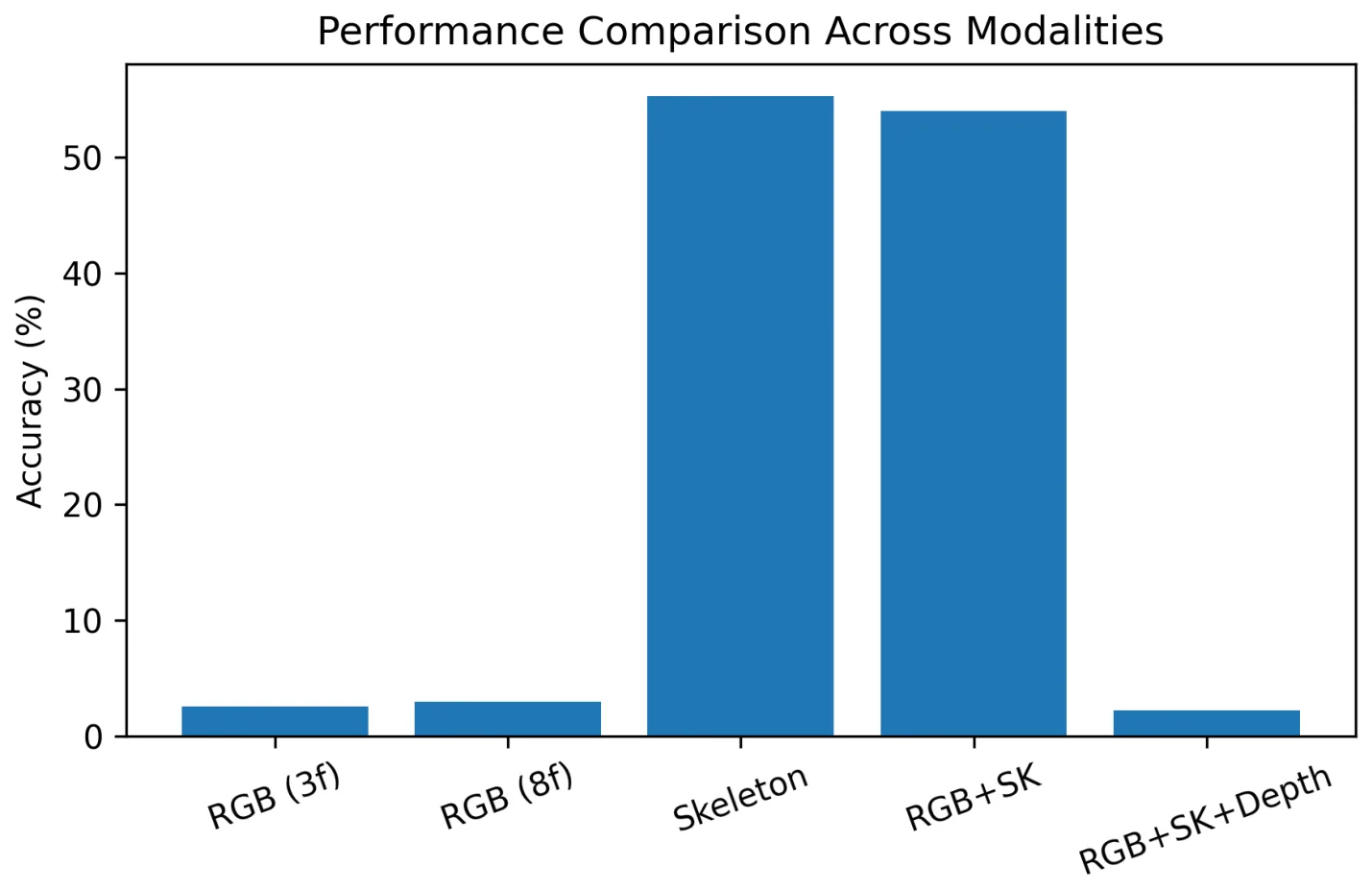
Recognition performance comparison across different modality configurations.

To provide an intuitive comparison, the recognition performance of different modality configurations is visualized in Figure 5.

Figure 5 further illustrates that increasing the number of modalities does not automatically lead to better recognition performance. Instead, performance improvements depend on whether additional modalities provide reliable and complementary information under the target recognition protocol.

It should be noted that the skeleton-only result in Table 2 represents a controlled reference within the modality-effectiveness evaluation framework. It is used to compare relative modality behavior under the same experimental conditions. In contrast, the final skeleton-based reference model reported in Table 3 is trained using the finalized skeleton preprocessing pipeline and ST-GCN-based recognition framework. Therefore, Table 2 evaluates relative modality effectiveness, whereas Table 3 reports the final recognition performance of the skeleton-based reference model.

Overall, the modality-effectiveness evaluation demonstrates that, under the evaluated cross-subject setting, the tested RGB and depth configurations do not consistently improve upon the skeleton-only configuration. These observations motivate the construction of the skeleton-based reference model and further analysis of fusion-induced performance changes.

### 3.2. Overall Performance of the Skeleton-Based Reference Model

Based on the modality-effectiveness evaluation results, skeleton representations are adopted as the primary reference modality for constructing the skeleton-based reference model under the evaluated cross-subject setting. The model adopts an ST-GCN-based architecture with learned confidence-weighted temporal pooling and a lightweight LayerNorm–Dropout–Linear classification head. The purpose is to evaluate the effectiveness of structural motion representations rather than to introduce additional architectural complexity. The overall performance on SLR500 is shown in Table 3.

As shown in Table 3, the skeleton-based reference model achieves 89.07% validation Top-1 accuracy, 86.37% test Top-1 accuracy, and 96.86% test Top-5 accuracy on SLR500. The difference between validation and test performance remains within an acceptable range, indicating that the model maintains effective recognition capability when evaluated on unseen signers. These results demonstrate that skeleton-based structural representations provide effective and relatively consistent information for cross-subject word-level sign language recognition under the evaluated protocol.

To further examine the applicability of the skeleton-based reference model beyond the primary SLR500 evaluation, an additional external validation experiment is conducted on AUTSL. This experiment focuses only on the skeleton-based reference model because its purpose is to provide supplementary validation of the identified reliable modality rather than to establish a complete cross-dataset multimodal benchmark. The results are summarized in Table 4.

As shown in Table 4, the skeleton-based reference model achieves 67.90% Test Top-1 accuracy and 92.97% Test Top-5 accuracy on AUTSL. Although the Top-1 accuracy decreases compared with SLR500, the model maintains relatively strong Top-5 recognition performance on an additional user-independent dataset. This result provides supplementary evidence that the skeleton-based reference model can maintain reasonable recognition performance beyond the primary evaluation dataset. It should be noted that AUTSL is used only for external validation and is not involved in modality-effectiveness comparison or model selection.

It should also be noted that the skeleton-only result in Table 2 belongs to the modality-effectiveness evaluation stage and is used to analyze the relative behavior of different modalities and fusion strategies under a controlled framework-level setting. In contrast, Table 3 reports the performance of the skeleton-based reference model trained with the finalized skeleton preprocessing pipeline and ST-GCN-based recognition framework. Therefore, these two results correspond to different experimental objectives and should not be directly interpreted as results from the same training stage. The subsequent analyses further investigate model behavior through ablation studies, sequence-length sensitivity analysis, stability analysis, convergence analysis, error-pattern analysis, and prediction transition analysis of fusion-induced performance changes.

### 3.3. Comparison with a Reproduced Representative Baseline Under the Same SLR500 Protocol

To further evaluate the competitiveness of the skeleton-based reference model under the target cross-subject setting, a representative pose/skeleton-based baseline is reproduced using the SL-GCN branch from the SAM-SLR-v2 implementation [26] and evaluated under the same SLR500 cross-subject protocol. This comparison uses the same dataset split and test set, and Top-1 and Top-5 accuracy are adopted as the evaluation metrics.

The reproduced SL-GCN baseline uses a 27-point pose representation, whereas the skeleton-based reference model in this study uses 25-joint skeleton features constructed from normalized joint coordinates, bone vectors, joint-angle information, and frame-level confidence scores. Both methods rely on structural motion representations rather than RGB appearance information. Therefore, this comparison provides a direct reference for evaluating pose- or skeleton-based recognition performance under the same cross-subject setting.

The results are summarized in Table 5.

As shown in Table 5, the reproduced SL-GCN baseline achieves 85.54% Test Top-1 accuracy and 96.45% Test Top-5 accuracy on the SLR500 cross-subject test set. Under the same evaluation protocol, the skeleton-based reference model achieves 86.37% Test Top-1 accuracy and 96.86% Test Top-5 accuracy. Compared with the reproduced SL-GCN baseline, the skeleton-based reference model improves Test Top-1 accuracy by 0.83 percentage points and Test Top-5 accuracy by 0.41 percentage points.

The performance gap is moderate because both methods are based on structural pose or skeleton representations, which already provide strong motion cues for cross-subject word-level sign language recognition. Nevertheless, the comparison is meaningful because it is conducted under the same SLR500 cross-subject protocol rather than relying on reported results from different datasets or evaluation settings. The result indicates that the skeleton-based reference model achieves competitive and slightly better test performance than a reproduced representative pose/skeleton-based baseline.

**Figure 6:**
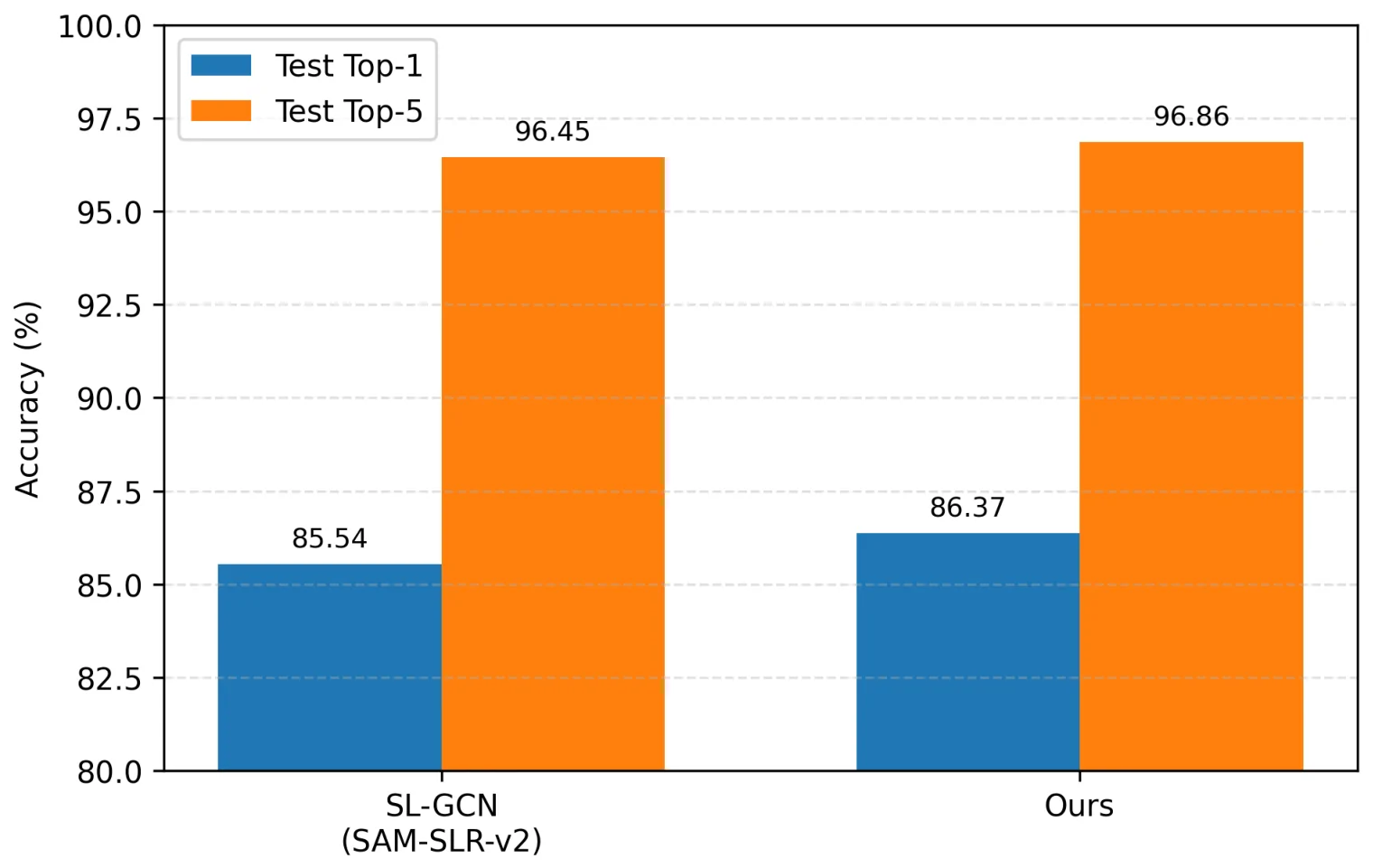
Same-protocol comparison between the reproduced SL-GCN baseline and the skeleton-based reference model on the SLR500 cross-subject test set.

The same-protocol comparison is visualized in Figure 6. The skeleton-based reference model shows slightly higher Test Top-1 and Test Top-5 accuracy than the reproduced SL-GCN baseline, suggesting that the adopted skeleton feature construction, learned confidence-weighted temporal pooling, and lightweight classification head provide additional benefit under the evaluated cross-subject setting.

Overall, this comparison provides a direct experimental reference for evaluating the skeleton-based reference model under the same SLR500 cross-subject protocol. The results do not imply broad superiority over all existing sign language recognition methods, but they demonstrate that the skeleton-based reference model remains competitive with a reproduced representative pose/skeleton-based baseline and achieves slightly better test-set recognition performance under the evaluated setting.

### 3.4. Ablation Study

To evaluate the contribution of key components in the skeleton-based reference model, replacement-based ablation experiments are conducted on the SLR500 cross-subject test set. The skeleton-based reference model consists of an ST-GCN encoder for spatio-temporal skeleton feature extraction, learned confidence-weighted temporal pooling for sequence aggregation, and a lightweight LayerNorm–Dropout–Linear classification head. Each ablation variant replaces one functional component of the reference model while keeping the input modality, preprocessing procedure, temporal length, training configuration, and evaluation metrics unchanged. The results are summarized in Table 6.

As shown in Table 6, the skeleton-based reference model achieves the best performance among all evaluated variants, obtaining 86.4% Top-1 accuracy and 96.9% Top-5 accuracy. Replacing the ST-GCN encoder with a TCN-only encoder decreases the Top-1 accuracy to 80.2% and the Top-5 accuracy to 93.5%. This result indicates that temporal convolution alone cannot fully capture the spatial dependency information among skeleton joints, highlighting the importance of graph-based topology modeling under the cross-subject ISLR setting.

The MLP+TCN encoder achieves 82.7% Top-1 accuracy and 94.8% Top-5 accuracy, which is better than the TCN-only variant but still lower than the ST-GCN reference model. Since this variant replaces graph convolution with frame-level feature projection while maintaining temporal modeling, the results suggest that explicit joint-relation modeling contributes to more effective skeleton representation learning.

The pooling and classifier replacement variants introduce smaller performance changes than the encoder replacements. Replacing learned confidence-weighted temporal pooling with max pooling reduces Top-1 accuracy from 86.4% to 85.1%, indicating that the adopted confidence-weighted temporal pooling provides a more effective aggregation strategy than max pooling under the evaluated setting. Replacing the LayerNorm–Dropout–Linear classification head with a single linear classifier decreases Top-1 accuracy to 85.7%, suggesting that the lightweight classification head provides additional performance improvement, although its contribution is smaller than that of the spatio-temporal encoder.

Overall, the replacement-based ablation results demonstrate that the ST-GCN encoder has the largest impact among the evaluated components, while the learned confidence-weighted temporal pooling and lightweight classification head provide additional improvements. These results support the use of the skeleton-based reference model for cross-subject word-level sign language recognition without introducing unnecessary multimodal fusion complexity.

### 3.5. Sequence Length Sensitivity Analysis

The temporal length of skeleton sequences affects both recognition performance and computational cost. A shorter sequence reduces computational complexity but may lose important motion variations, whereas a longer sequence preserves more temporal information but introduces additional computational overhead.

To investigate the influence of temporal length selection, a sequence-length sensitivity analysis is conducted under the same SLR500 cross-subject protocol. The model architecture, preprocessing strategy, training configuration, and random seed are kept unchanged, and only the temporal length $T_{s}$ is varied. Three temporal lengths, including 32, 48, and 64 frames, are evaluated. The results are summarized in Table 7.

As shown in Table 7, increasing the temporal length from 32 to 64 frames improves the Test Top-1 accuracy from 84.05% to 86.37%. Compared with the 32-frame setting, the 64-frame setting achieves a 2.32 percentage-point improvement, indicating that shorter temporal inputs may not preserve sufficient motion evolution information for distinguishing fine-grained sign categories.

The improvement in Top-1 accuracy is accompanied by increased computational cost. When $T_{s}$ increases from 32 to 64, the computational cost increases from 4.24 to 8.47 GMACs per sample. This indicates that longer sequences provide additional temporal information at the cost of increased computation.

However, Top-5 accuracy does not monotonically increase with temporal length. The 48-frame setting achieves the highest Top-5 accuracy of 97.11%, while the 64-frame setting provides the best Top-1 accuracy. Therefore, $T_{s}=64$ is selected as the default temporal length for the final skeleton-based reference model because it achieves the highest exact classification accuracy among the evaluated settings.

Overall, the sequence-length sensitivity analysis shows that temporal length selection affects the balance between recognition accuracy and computational cost. Within the evaluated range, the 64-frame setting provides the best Top-1 recognition performance.

### 3.6. Stability Analysis

To evaluate the robustness and reproducibility of the skeleton-based reference model under different random initializations, three independent training runs are conducted using random seeds 42, 123, and 3407. All runs use the same dataset split, preprocessing pipeline, model architecture, temporal length, and optimization configuration. For each run, the checkpoint with the highest validation Top-1 accuracy is selected, and the corresponding model is evaluated on the SLR500 cross-subject test set containing 12,162 samples.

The stability results are summarized in Table 8. In addition to the results of individual runs, the mean, standard deviation, and performance range are reported to describe the variation across different random seeds.

As shown in Table 8, the skeleton-based reference model maintains consistent performance across different random seeds. The mean Test Top-1 accuracy is 86.15%, with a standard deviation of 1.00%, while the mean Test Top-5 accuracy is 96.89%, with a standard deviation of 0.02%. The performance range is 1.96 percentage points for Test Top-1 accuracy and 0.04 percentage points for Test Top-5 accuracy.

These results suggest that the observed performance is not highly sensitive to random initialization. Although the Test Top-1 accuracy shows moderate variation across runs, the overall recognition performance remains stable under the SLR500 cross-subject evaluation protocol. Therefore, the multi-seed results provide additional support for the reproducibility of the skeleton-based reference model.

To further examine prediction-level consistency across random seeds, pairwise prediction agreement is computed between each pair of independently trained models. The results are summarized in Table 9.

As shown in Table 9, the prediction agreement between different seed pairs remains above 86%. This indicates that independently trained models produce similar prediction patterns on most test samples, further supporting the stability of the skeleton-based reference model at the prediction level.

In addition, the test samples are divided into three groups according to the prediction agreement patterns across the three random seeds. The results are shown in Table 10.

As shown in Table 10, 82.78% of the test samples receive the same prediction from all three models, and this subset achieves 95.34% mean single-seed accuracy. In contrast, samples with two-seed agreement or complete disagreement show much lower mean single-seed accuracy. This indicates that prediction inconsistency across random seeds is associated with more difficult or ambiguous samples.

Overall, the stability analysis shows that the skeleton-based reference model achieves reproducible performance across random initializations. The prediction-level consistency results further suggest that most test samples lead to stable predictions, while the inconsistent subset reflects higher recognition uncertainty under the cross-subject setting.

**Figure 7:**
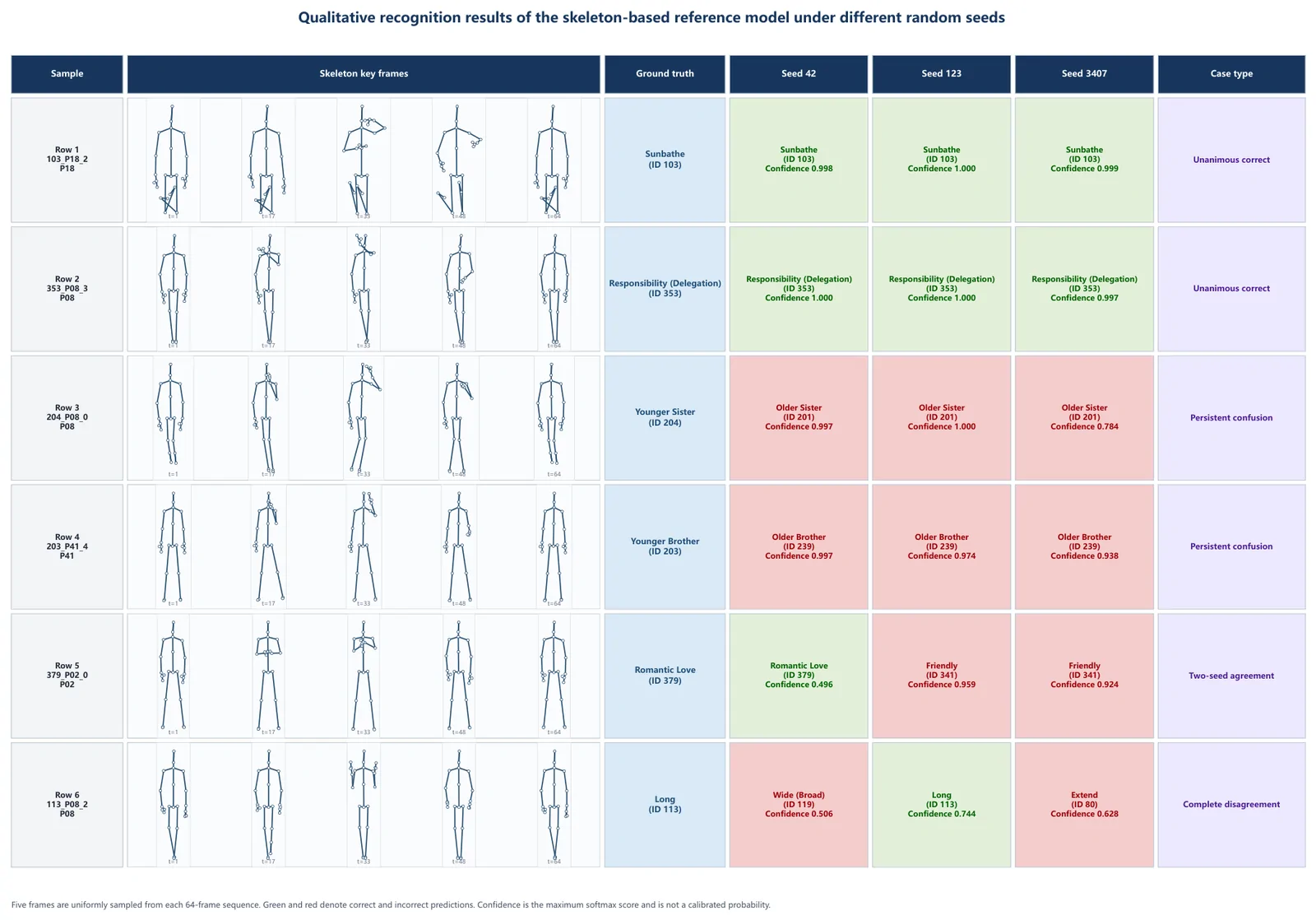
Qualitative prediction consistency analysis of the skeleton-based reference model under different random seeds. Green and red indicate correct and incorrect predictions, respectively. Confidence denotes the maximum softmax score and is not a calibrated probability.

To further provide an intuitive understanding of prediction consistency across different random seeds, qualitative examples are presented in Figure 7. The selected cases include unanimous correct predictions, persistent confusion cases, two-seed agreement cases, and complete disagreement cases. These examples demonstrate that samples with consistent predictions across different random seeds usually correspond to clear motion patterns, whereas samples with prediction variations often correspond to visually similar signs or more challenging motion patterns.

These qualitative observations also provide a transition toward the subsequent error-pattern analysis, where class-level ambiguity and signer-related variations are further investigated.

### 3.7. Statistical Significance Analysis

To further evaluate whether the observed performance differences between the skeleton-based reference model and the fusion model are statistically meaningful, a paired McNemar test is conducted based on sample-level prediction outcomes.

Since both models are evaluated on the same test samples, McNemar’s test is appropriate for comparing paired classification predictions. Let *b* denote the number of samples correctly classified by the skeleton-based reference model but incorrectly classified by the fusion model, and let *c* denote the number of samples incorrectly classified by the skeleton-based reference model but correctly classified by the fusion model.

The McNemar statistic is calculated as

(36) $$\chi^{2}=\frac{{\left(|b-c|-1\right)}^{2}}{b+c}.$$

For the comparison between the skeleton-based reference model and the RGB–Skeleton MAGF fusion model, b=4558 and c=259. The resulting statistic is

(37) $$\chi^{2}=3834.71,$$

with

(38) p<0.001.

The result indicates that the prediction behaviors of the two models are significantly different under the same cross-subject test samples. This statistical analysis supports the observation that the evaluated fusion strategy introduces substantial prediction changes compared with the skeleton-based reference model.

### 3.8. Convergence Analysis

**Figure 8:**
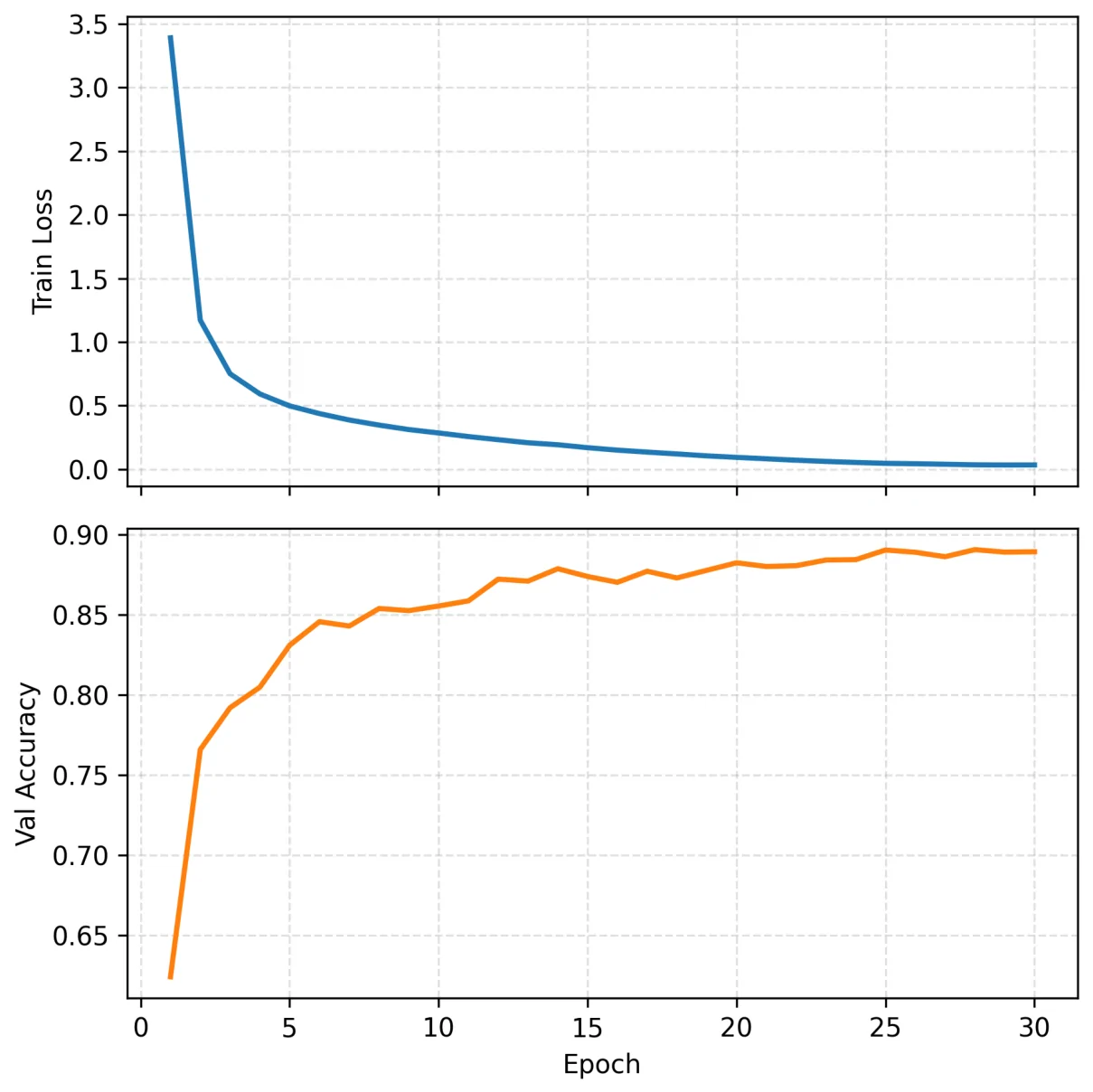
Training loss and validation accuracy curves of the skeleton-based reference model.

To investigate the optimization behavior of the skeleton-based reference model, the training loss and validation accuracy are recorded during training. The results are shown in Figure 8.

As shown in Figure 8, the training loss decreases rapidly during the early training stage and gradually converges as training proceeds. Meanwhile, the validation accuracy increases during the early stage and remains relatively stable in later training stages. This optimization behavior indicates that the model can effectively learn discriminative skeleton representations while maintaining stable validation performance.

The decreasing training loss is accompanied by increasing and later stabilized validation accuracy. No obvious collapse of validation accuracy is observed after convergence, suggesting that no obvious overfitting pattern is observed under the current training configuration. Combined with the multi-seed stability analysis, the convergence results provide additional evidence that the skeleton-based reference model can be trained in a stable manner under the SLR500 cross-subject protocol.

### 3.9. Error Pattern Analysis

**Figure 9:**
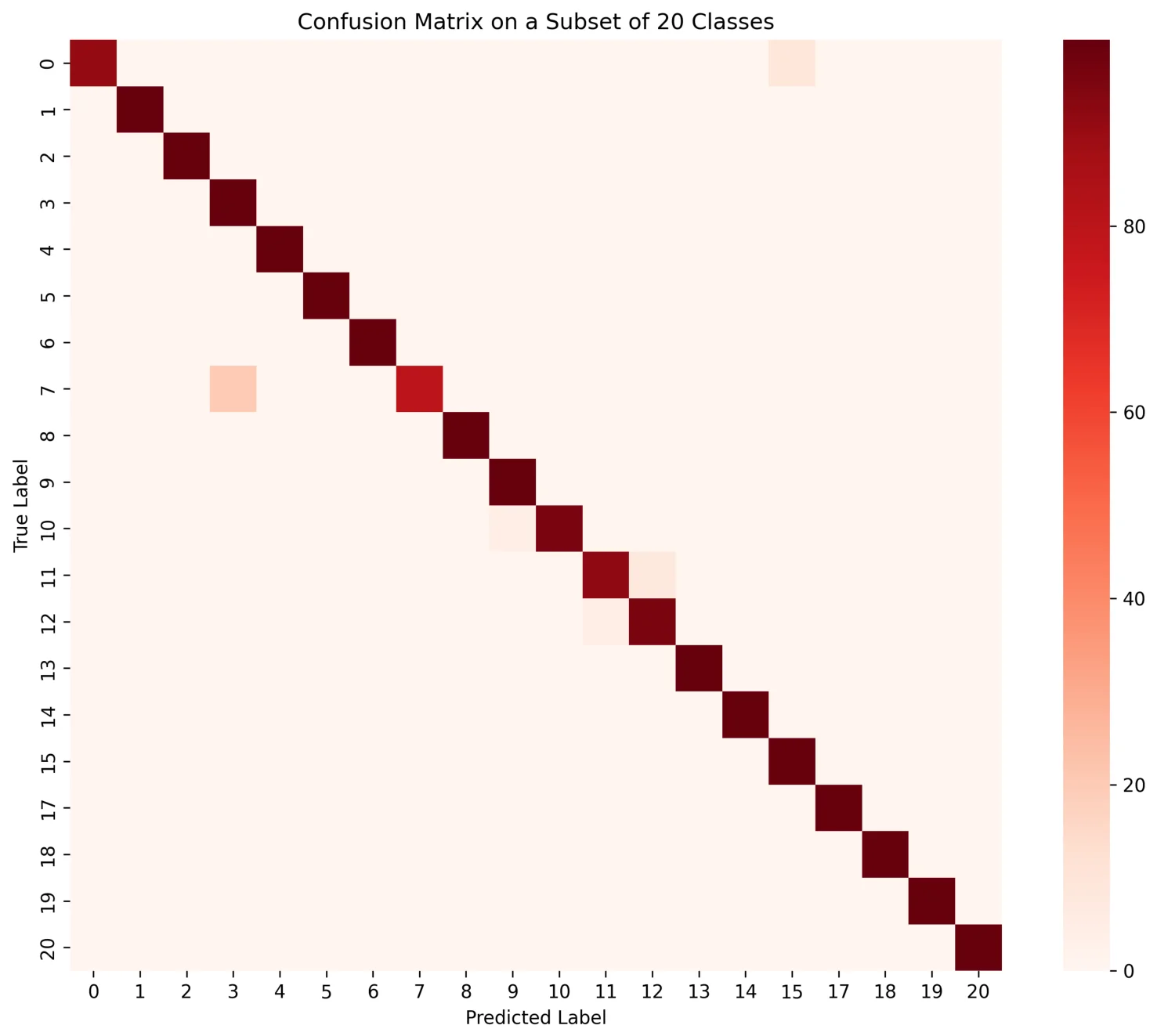
Confusion matrix on a selected subset of 20 classes.

Since SLR500 contains 500 sign categories, visualizing the complete confusion matrix in the main text would reduce interpretability. Therefore, a selected subset of 20 classes is used for visualization, and the corresponding confusion matrix is presented in Figure 9. To provide a more comprehensive analysis of recognition errors, subject-level performance, error-prone classes, and persistent confusion patterns across random seeds are further investigated.

As shown in Figure 9, most predictions in the selected subset are concentrated along the diagonal, indicating that the skeleton-based reference model can effectively distinguish many sign categories using structural motion information. However, several off-diagonal responses remain, particularly for categories with similar motion trajectories or categories requiring fine-grained local hand and facial cues. These observations indicate that skeleton representations are effective for capturing global body motion patterns but may have limited ability to represent appearance-dependent local information.

To further analyze recognition errors, prediction results on the SLR500 test set are statistically summarized. Among $N_{\mathrm{test}}=12,162$ test samples, $N_{\mathrm{err}}=1658$ samples are misclassified. The overall test error rate is defined as

(39) $$E_{\mathrm{test}}=\frac{N_{\mathrm{err}}}{N_{\mathrm{test}}},$$

where $N_{\mathrm{test}}$ represents the number of test samples and $N_{\mathrm{err}}$ denotes the number of incorrect predictions. Substituting the observed values gives $E_{\mathrm{test}}\approx 13.63\%$. The distribution of recognition errors is further analyzed from subject-level and class-level perspectives.

As shown in Table 11, recognition performance varies among unseen test signers. P18 achieves the highest mean Top-1 accuracy of 91.85%, whereas P02 achieves the lowest mean Top-1 accuracy of 77.65%. The difference of 14.20 percentage points suggests that cross-subject recognition performance is influenced by signer-specific factors, including motion style, execution variation, and possible skeleton estimation differences. The relatively small standard deviations across random seeds indicate that these subject-level differences are mainly related to signer characteristics rather than random initialization.

Furthermore, the subject macro-average accuracy remains close to the sample micro-average accuracy across different seeds. For example, seed 42 achieves 86.39% subject macro-average accuracy and 86.37% sample micro-average accuracy. This indicates that the overall recognition performance is not dominated by a specific test subject.

Class-level error statistics are summarized in Table 12.

Table 12 summarizes classes with frequent recognition errors. These categories are mainly associated with local hand–face interactions, subtle hand-shape differences, color-related semantics, or visually similar motion patterns. Since each category contains a limited number of test samples, these results are interpreted as indicators of potentially challenging categories rather than definitive estimates of class difficulty.

To investigate whether confusion patterns are consistent across different random seeds, persistent high-frequency confusion pairs are summarized in Table 13.

Table 13 summarizes confusion pairs that repeatedly appear across three random seeds. The total count is calculated by summing the misclassification occurrences of the three independently trained models, rather than counting unique test samples. The repeated occurrence of these pairs indicates that these categories tend to be consistently confused under the skeleton-based representation.

Many confusion pairs involve kinship terms, semantically related categories, or visually similar motion patterns, such as younger sister versus older sister, younger brother versus older brother, real versus true, and son versus daughter. These results suggest that skeleton trajectories can effectively capture global motion structures but may remain insufficient for distinguishing categories that depend on subtle hand-shape, facial-region, or local semantic cues.

Overall, the error-pattern analysis indicates that the remaining recognition errors are structured rather than purely random. These errors are mainly associated with unseen-signer variation, class-level ambiguity, and the limited ability of skeleton representations to capture fine-grained local visual information. Future improvements may therefore focus on enhancing local hand and facial representations or developing more reliable motion-aware representations, rather than simply increasing model complexity.

### 3.10. Analysis of Fusion-Induced Performance Changes

The modality-effectiveness evaluation shows that the tested fusion configurations do not outperform the skeleton-only configuration under the unified cross-subject setting. However, overall accuracy differences alone cannot explain how fusion changes individual sample predictions. Therefore, this section analyzes fusion-induced performance changes from three complementary perspectives. First, the overall error rates of different modality configurations are compared to measure global performance variation. Second, sample-level prediction transitions are analyzed to investigate whether fusion corrects samples that are misclassified by the skeleton-based reference model or introduces additional errors into samples that are originally recognized correctly. Third, the learned modality contribution weights of the MAGF module are visualized to examine how the fusion model assigns relative contributions to RGB and skeleton modalities.

It should be noted that these analyses correspond to different experimental purposes. The error-rate comparison is conducted using the modality-effectiveness evaluation stage, where different modality configurations are compared under a controlled framework-level setting. In contrast, the sample-level transition analysis and MAGF visualization are based on the final fusion model predictions and are used to further analyze prediction changes and modality weighting behavior.

Following the error-rate definition in Equation (35), the error-rate changes of different fusion configurations are summarized in Table 14.

As shown in Table 14, the evaluated RGB–Skeleton fusion configurations increase the error rate by approximately 1.3–2.0 percentage points compared with the skeleton-only configuration during the modality-effectiveness evaluation stage. When depth is additionally introduced, the RGB–Skeleton–Depth configuration exhibits a substantially larger error increase. These results indicate that the evaluated auxiliary modalities do not provide consistent global performance improvements under the current cross-subject setting. However, overall error-rate comparison alone cannot reveal how individual predictions are changed after fusion.

To further investigate prediction-level changes, the predictions of the final skeleton-based reference model and the evaluated RGB–Skeleton MAGF fusion model are compared on the same SLR500 test set. The transition statistics are summarized in Table 15.

To quantify the sample-level influence of fusion, the additional-error rate and correction rate are defined as

(40) $$R_{\mathrm{add}}=\frac{|T_{degraded}|}{|D_{test}|},\;R_{\mathrm{corr}}=\frac{|T_{corrected}|}{|D_{test}|}.$$

The net prediction change caused by fusion is further defined as

(41) $$\Delta_{\mathrm{fusion}}=R_{\mathrm{add}}-R_{\mathrm{corr}}.$$

A positive $\Delta_{\mathrm{fusion}}$ indicates that the evaluated fusion model introduces more newly incorrect predictions than corrected predictions under the current setting.

As shown in Table 15, the prediction transitions exhibit a clear asymmetric pattern. Among the 12,162 test samples, 4558 samples are correctly classified by the skeleton-based reference model but become incorrect after fusion, whereas only 259 samples are corrected by the RGB–Skeleton MAGF fusion model after being misclassified by the skeleton-based reference model. According to Equation (40), $R_{\mathrm{add}}=37.48\%$ and $R_{\mathrm{corr}}=2.13\%$. Therefore, Equation (41) gives $\Delta_{\mathrm{fusion}}=35.35$ percentage points.

This result indicates that, under the evaluated setting, the RGB–Skeleton MAGF fusion model does not primarily improve the difficult samples of the skeleton-based reference model. Instead, it introduces additional prediction errors into a considerable number of samples that can already be correctly recognized by the skeleton-based reference model.

**Figure 10:**
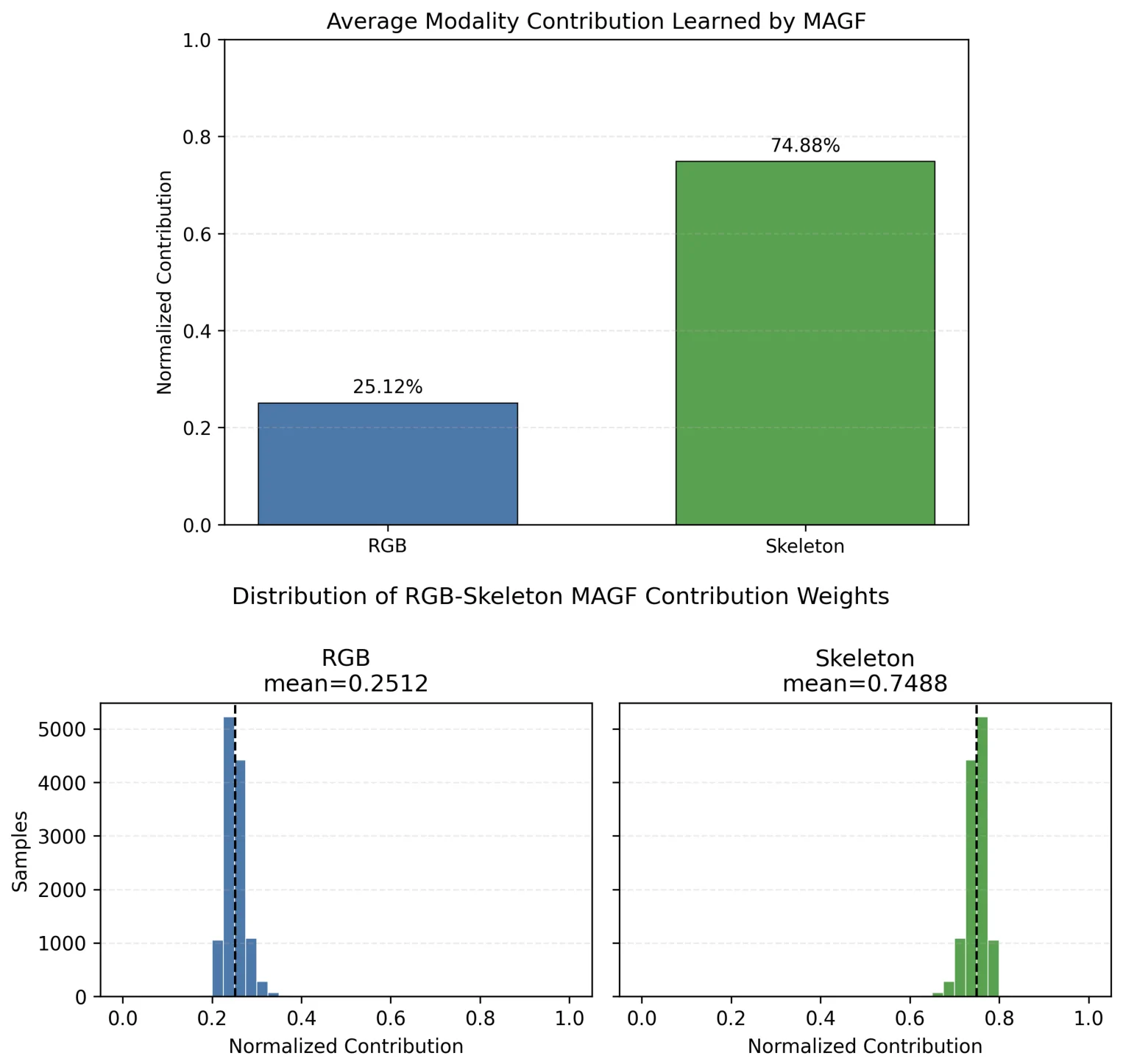
Visualization of modality contribution weights learned by the RGB–Skeleton MAGF fusion model. The upper panel presents the average normalized contribution of each modality, while the lower panel presents the distribution of sample-level contribution weights.

To further analyze the behavior of the MAGF fusion module, the normalized modality contribution weights learned by the RGB–Skeleton MAGF model are visualized in Figure 10. The weights are normalized between RGB and skeleton modalities and are used to analyze the relative contribution assigned by the fusion mechanism.

As shown in Figure 10, the MAGF module assigns a larger average normalized contribution to the skeleton modality than to the RGB modality. The average contribution of skeleton is 74.88%, while RGB contributes 25.12%. The distribution of sample-level weights also shows a consistent skeleton-preferred pattern, indicating that the fusion module tends to assign higher relative importance to skeleton representations under the evaluated setting.

However, this weighting behavior does not translate into improved recognition accuracy over the skeleton-only configuration, because the RGB–Skeleton MAGF fusion model still performs lower than the skeleton-based reference model in the modality-effectiveness evaluation. Therefore, the visualization suggests that emphasizing the modality with stronger recognition capability under the evaluated setting is not sufficient to guarantee effective multimodal complementarity when auxiliary modalities provide limited additional discriminative information.

In this study, the observed fusion-induced degradation is described as negative-transfer-like behavior only at the empirical prediction level. Specifically, it refers to the situation where the evaluated fusion model introduces more newly incorrect predictions than corrected predictions compared with the skeleton-based reference model. This observation does not constitute a strict proof of internal negative transfer mechanisms or feature-level interference among modalities. Instead, it provides empirical evidence that auxiliary modalities should be carefully evaluated before being incorporated into multimodal fusion systems.

Overall, the error-rate comparison, sample-level prediction transition analysis, and MAGF weight visualization consistently indicate that the evaluated fusion configurations do not provide stable performance improvements over the skeleton-based reference model under the current cross-subject setting. These findings further support the use of the skeleton-based reference model for reliability-oriented modality analysis under the evaluated cross-subject setting.

## 4. Discussion

### 4.1. Modality Effectiveness Under Cross-Subject Settings

The experimental results show that different modality configurations exhibit different effectiveness under the SLR500 cross-subject word-level sign language recognition setting. Under the evaluated RGB configurations, RGB-only models achieve limited recognition performance, whereas the skeleton-only configuration provides more discriminative structural information in the modality-effectiveness evaluation stage. The final skeleton-based reference model further demonstrates that skeleton representations can support effective recognition performance when combined with an ST-GCN encoder, learned confidence-weighted temporal pooling, and a lightweight classification head. These results indicate that, under cross-subject conditions, the recognition performance is strongly influenced by the reliability, stability, and task suitability of different modalities.

The skeleton modality represents human body motion through structured keypoint sequences, where the extracted features mainly describe relative joint relationships and temporal motion trajectories. Compared with RGB appearance features, skeleton representations reduce part of the influence caused by background, illumination, clothing, viewpoint, and signer appearance variations, allowing the model to focus more on structural motion patterns. In the cross-subject setting, training and testing signers are completely separated, and different individuals may exhibit variations in body shape, motion amplitude, signing style, and acquisition conditions. However, the structural characteristics of the same sign may still maintain a certain degree of consistency across unseen signers. Therefore, skeleton representations based on relative geometry and temporal dynamics are more likely to capture signer-robust motion patterns under the evaluated protocol.

Before ST-GCN, graph representation learning has been explored through spectral graph convolution and graph attention mechanisms [19,20,21]. ST-GCN further extends these ideas to skeleton-based action recognition by modeling human joints as spatio-temporal graphs [22]. In this study, the skeleton modality achieves better performance than the evaluated RGB configurations in the modality-effectiveness analysis and provides a stable reference for the final recognition model. These results support the effectiveness of structured skeleton representations for cross-subject word-level sign language recognition under the current evaluation setting.

In contrast, RGB-based recognition mainly relies on visual appearance and motion information extracted from video frames. Under cross-subject settings, variations in background, illumination, clothing, body appearance, and viewpoint may introduce distribution differences between training and testing samples, increasing the difficulty of learning consistent visual representations [4,5,6]. Furthermore, sign language recognition often depends on fine-grained hand movements and local facial cues, while these regions may be affected by occlusion, motion blur, limited resolution, and insufficient temporal sampling. The key-frame sampling strategy adopted in this study reduces computational cost but may also limit the preservation of complete temporal dynamics. Therefore, the evaluated RGB configurations do not provide stable recognition benefits under the current cross-subject protocol, rather than indicating that RGB information is generally ineffective for sign language recognition.

It should be emphasized that the above observation does not imply that RGB information has limited value in all sign language recognition scenarios. RGB videos can provide important appearance-related information, including hand shape, facial expression, and visual context, when high-quality inputs, effective temporal modeling, and appropriate feature-extraction strategies are available. Instead, the findings of this study indicate that, under the evaluated SLR500 cross-subject setting, skeleton representations provide a more stable reference modality than the tested RGB configurations.

The same-protocol comparison with the reproduced SL-GCN branch from SAM-SLR-v2 further supports the effectiveness of structural representations under the SLR500 cross-subject setting. Both the reproduced SL-GCN baseline and the skeleton-based reference model rely on pose or skeleton representations and are evaluated under the same SLR500 cross-subject protocol. The reproduced SL-GCN baseline achieves 85.54% Test Top-1 accuracy, while the skeleton-based reference model achieves 86.37%. The improvement is moderate and should not be interpreted as broad superiority over existing sign language recognition methods. Instead, it indicates that the skeleton-based reference model remains competitive with a representative pose/skeleton-based baseline under the same protocol.

Overall, the results suggest that cross-subject word-level sign language recognition performance does not automatically improve by increasing the number of modalities. Instead, the effectiveness of a modality depends on its information quality, representation stability, and compatibility with the target recognition scenario. Under the evaluated protocol, skeleton representations provide relatively consistent structural motion cues and are therefore suitable as a reliable reference modality for modality-effectiveness analysis and cross-subject recognition.

### 4.2. Condition-Dependent Effectiveness of Multimodal Fusion

Multimodal fusion is commonly based on the assumption that different modalities provide complementary information from appearance, structure, and spatial perspectives, thereby improving recognition performance [26,33]. However, the effectiveness of multimodal fusion is highly dependent on whether the involved modalities provide sufficiently reliable, discriminative, and compatible information under the target evaluation protocol. When auxiliary modalities contain limited complementary information or exhibit inconsistent representation characteristics, direct fusion may not necessarily lead to additional recognition gains.

The modality-effectiveness evaluation results show that the evaluated RGB–Skeleton fusion configurations do not outperform the skeleton-only reference model under simple fusion, MAGF, or residual fusion strategies. Furthermore, introducing depth information leads to a substantial performance decrease in the RGB–Skeleton–Depth configuration. These results indicate that, under the current SLR500 cross-subject setting, the evaluated RGB and depth configurations do not provide stable complementary benefits for the skeleton-based reference model.

From the perspective of multimodal representation learning, fusion performance is influenced by modality quality, representation consistency, alignment relationship, and fusion strategy [33]. Previous studies on transfer learning and multi-task learning have also shown that auxiliary information may not always improve the target task when the relationship between source and target information is insufficiently aligned [37,38]. In this study, the skeleton modality provides relatively consistent structural motion information, whereas the evaluated RGB and depth configurations provide limited additional gains under the current protocol. Therefore, the observed performance degradation may be related to insufficient complementary information between modalities, rather than indicating that multimodal fusion itself is ineffective.

The sample-level prediction transition analysis further provides empirical evidence of fusion-induced performance changes. Compared with the skeleton-based reference model, the evaluated fusion model introduces a larger number of newly incorrect predictions than corrected predictions. This asymmetric transition pattern indicates that fusion does not consistently improve difficult samples and may introduce additional uncertainty into samples that are already correctly recognized by the skeleton-based reference model.

In this study, such behavior is described as negative-transfer-like behavior only at the prediction level. Specifically, it refers to the observation that the evaluated fusion configuration produces more additional recognition errors than corrected cases under the current cross-subject setting. This observation does not constitute a strict proof of feature-level negative transfer, gradient conflicts, or internal modality interference mechanisms. Instead, it provides empirical evidence that auxiliary modalities should be evaluated carefully before being incorporated into multimodal fusion systems.

The MAGF weight visualization further provides insight into the behavior of the fusion module. As shown in Figure 10, the RGB–Skeleton MAGF model assigns a higher average normalized contribution to the skeleton modality than to the RGB modality, with 74.88% for skeleton and 25.12% for RGB. The sample-level weight distribution also shows a consistent preference toward skeleton representations under the evaluated setting. This indicates that the gating mechanism can adaptively adjust the relative contribution of different modalities based on the learned representations.

However, the observed weighting preference does not directly translate into improved recognition performance. Although MAGF assigns higher relative importance to the skeleton modality, the RGB–Skeleton MAGF configuration still does not outperform the skeleton-only reference model. This suggests that adaptive weighting can regulate modality contribution, but it cannot guarantee effective complementarity when auxiliary modalities provide limited additional discriminative information.

Therefore, the findings of this study do not indicate that multimodal fusion is universally ineffective. Instead, they demonstrate that fusion effectiveness is condition-dependent. Under scenarios with higher-quality RGB inputs, more accurate hand and facial representations, more reliable depth information, stronger temporal modeling, or large-scale pretraining, multimodal fusion may still provide valuable complementary information. However, under the current SLR500 cross-subject protocol, reliability-oriented modality selection is more important than simply increasing the number of modalities or introducing more complex fusion mechanisms.

### 4.3. Limitations

Although this study establishes a skeleton-based reference model through modality-effectiveness analysis and achieves stable performance under the SLR500 cross-subject setting, several limitations remain.

First, skeleton representations mainly describe global body topology and joint motion trajectories, but they have limited ability to capture fine-grained hand shapes, facial cues, and local hand–face interactions. The error-pattern analysis shows that several difficult categories are related to subtle local differences and visually similar motion patterns. Therefore, future work may incorporate more reliable hand keypoints, facial representations, or local region features to improve fine-grained sign discrimination.

Second, the primary modality-effectiveness analysis is conducted on SLR500, while AUTSL is used as an external validation dataset for the skeleton-based reference model. However, complete multimodal evaluation under unified cross-dataset protocols remains limited. Different datasets may vary in vocabulary size, signer distribution, acquisition conditions, and modality quality. Therefore, broader validation on additional datasets and more consistent multimodal evaluation protocols is still required.

Third, the analysis of fusion-induced performance degradation is mainly based on accuracy changes, sample-level prediction transitions, and MAGF contribution visualization. These analyses provide empirical evidence of fusion behavior under the evaluated setting, but they do not fully explain the internal feature interaction mechanisms between modalities. Future work will further investigate modality alignment, feature-level interaction, and optimization dynamics to better understand multimodal fusion behavior.

Finally, this study does not conclude that RGB, depth, or multimodal fusion are ineffective for sign language recognition. Instead, the results indicate that fusion effectiveness depends on modality quality, representation reliability, and compatibility with the target evaluation protocol. Future research may explore stronger visual representations, large-scale pretraining, and more adaptive fusion strategies to further improve cross-subject sign language recognition.

## 5. Conclusions

This study investigates modality effectiveness in cross-subject word-level sign language recognition. Using SLR500 as the primary dataset, RGB, skeleton, depth, and several fusion configurations are systematically evaluated under a unified cross-subject protocol. The experimental results show that the evaluated RGB and depth configurations do not provide stable complementary gains over skeleton representations under the current setting. In particular, the tested RGB–Skeleton and RGB–Skeleton–Depth fusion configurations do not outperform the skeleton-only reference. These findings indicate that, in cross-subject word-level sign language recognition, increasing the number of modalities does not necessarily improve performance. Instead, the discriminative quality, reliability, and compatibility of each modality are more critical.

The analysis of fusion-induced performance changes further shows that the evaluated fusion configurations introduce additional recognition errors compared with the skeleton-based reference. The sample-level prediction transition analysis indicates that many samples correctly classified by the skeleton-based reference model become incorrect after fusion, whereas only a limited number of samples are corrected by the fusion model. These results suggest that, under the evaluated setting, auxiliary modalities should not be assumed to be beneficial without verifying their reliability, alignment, and compatibility under the target evaluation protocol.

Based on the modality-effectiveness analysis, this study establishes a skeleton-based reference model for cross-subject word-level sign language recognition. The model uses skeleton sequences as input, extracts spatial joint topology and temporal motion dynamics through an ST-GCN encoder, aggregates frame-level features using learned confidence-weighted temporal pooling, and performs classification with a lightweight LayerNorm–Dropout–Linear head. The model achieves 86.37% Test Top-1 accuracy and 96.86% Test Top-5 accuracy on the SLR500 cross-subject test set. A same-protocol comparison with the reproduced SL-GCN branch from SAM-SLR-v2 further shows that the skeleton-based reference model achieves competitive and slightly better test performance than a representative pose/skeleton-based baseline under the SLR500 cross-subject setting. On the external AUTSL validation dataset, the skeleton-based reference model achieves 67.90% Test Top-1 accuracy and 92.97% Test Top-5 accuracy. Ablation experiments, multi-seed stability analysis, sequence-length sensitivity analysis, convergence analysis, and error-pattern analysis further support the reliability of skeleton representations under the evaluated cross-subject settings.

This study still has several limitations. Skeleton representations have limited ability to describe fine-grained hand shapes, facial cues, and local hand–face interactions, which may lead to confusion between structurally or semantically similar classes. In addition, the primary modality-effectiveness analysis is conducted on SLR500, while AUTSL is used only for external skeleton-based validation rather than complete cross-dataset multimodal benchmarking. The analysis of fusion-induced degradation is also based mainly on overall performance changes, sample-level prediction transitions, and MAGF weight visualization, rather than a complete mechanistic explanation of cross-modal feature interactions.

Future work will further investigate more reliable hand and facial representations, feature-space and gradient-level fusion behavior, broader cross-dataset validation, and multimodal fusion strategies under higher-quality visual inputs and large-scale pretraining conditions. Overall, the main conclusion of this study is that multimodal fusion in sign language recognition should be treated as condition-dependent rather than automatically beneficial. When auxiliary modalities are unstable, weakly discriminative, or poorly aligned with the target protocol, reliability-oriented modality selection may be more effective than simply increasing the number of modalities or introducing more complex fusion structures.

## Figures and Tables

**Table 1:** Computational cost of the skeleton-based reference model.

Metric	Value
Trainable Parameters	5.38 M
MACs	8.47 GMACs/sample
Peak Training Memory	Approximately 864 MB

**Table 2:** Modality-effectiveness evaluation results under the cross-subject setting.

Configuration	Input Modality	Setting	Top-1 Accuracy (%)
RGB-only	RGB	3 frames	2.6
RGB-only	RGB	8 frames	3.0
Skeleton-only	Skeleton	–	**55.3**
RGB–Skeleton	RGB + Skeleton	Simple fusion	54.0
RGB–Skeleton	RGB + Skeleton	MAGF	54.0
RGB–Skeleton–Depth	RGB + Skeleton + Depth	MAGF	2.24
RGB–Skeleton	RGB + Skeleton	Residual, 3 frames	53.3
RGB–Skeleton	RGB + Skeleton	Residual, 8 frames	54.0
RGB–Skeleton	RGB + Skeleton	Residual, 16 frames	51.3

*Note:* Bold indicates the highest Top-1 accuracy among the evaluated configurations.

**Table 3:** Overall performance of the skeleton-based reference model on SLR500.

Metric	Value
Validation Top-1 Accuracy	89.07%
Test Top-1 Accuracy	86.37%
Test Top-5 Accuracy	96.86%
Test Error Rate	13.63%

**Table 4:** External validation of the skeleton-based reference model on SLR500 and AUTSL.

Dataset	Modality	Test Top-1 Accuracy (%)	Test Top-5 Accuracy (%)
SLR500	Skeleton	86.37	96.86
AUTSL	Skeleton	67.90	92.97

**Table 5:** Comparison with a reproduced representative baseline under the same SLR500 cross-subject protocol.

Method	Structural Input	Test Top-1 (%)	Test Top-5 (%)
SL-GCN from SAM-SLR-v2	27-point pose representation	85.54	96.45
Skeleton-based reference model	25-joint skeleton features	**86.37**	**96.86**

*Note:* Bold indicates the best performance in each metric column.

**Table 6:** Replacement-based ablation study of the skeleton-based reference model on the SLR500 cross-subject test set.

Setting	Modification	Top-1 Accuracy (%)	Top-5 Accuracy (%)
Reference model	ST-GCN + confidence-weighted pooling + lightweight classifier	**86.4**	**96.9**
TCN-only encoder	ST-GCN → Temporal Conv1D	80.2	93.5
MLP + TCN encoder	Graph convolution → MLP + Temporal Conv1D	82.7	94.8
Max pooling	Confidence-weighted pooling → Max pooling	85.1	96.2
Linear classifier	LN–Dropout–Linear → Linear	85.7	96.5

*Note:* Bold indicates the best performance in each accuracy column.

**Table 7:** Sequence-length sensitivity analysis of the skeleton-based reference model.

$T_{s}$	Val. Top-1 (%)	Test Top-1 (%)	Test Top-5 (%)	MACs (G/Sample)
32	86.19	84.05	96.77	4.24
48	87.12	84.97	**97.11**	6.35
64	**89.07**	**86.37**	96.86	8.47

*Note:* Bold indicates the best performance in each accuracy column.

**Table 8:** Stability analysis of the skeleton-based reference model under different random seeds.

Seed	Validation Top-1 (%)	Test Top-1 (%)	Test Top-5 (%)
42	89.07	86.37	96.86
123	89.36	87.02	96.90
3407	87.42	85.06	96.90
Mean ± SD	88.62 ± 1.04	86.15 ± 1.00	96.89 ± 0.02
Range	1.94	1.96	0.04

**Table 9:** Pairwise prediction agreement between models trained with different random seeds.

Seed Pair	Prediction Agreement (%)
42 vs. 123	88.52
42 vs. 3407	86.58
123 vs. 3407	86.92

**Table 10:** Prediction consistency across three random seeds on the SLR500 test set.

Prediction Pattern	Percentage (%)	Mean Single-Seed Accuracy (%)
Unanimous agreement	82.78	95.34
Two-seed agreement	13.67	47.42
Complete disagreement	3.54	20.80

**Table 11:** Subject-level Top-1 accuracy across different random seeds.

Test Subject	Seed 42 Top-1 (%)	Seed 123 Top-1 (%)	Seed 3407 Top-1 (%)	Mean ± SD (%)
P02	77.04	78.39	77.53	77.65 ± 0.68
P08	86.82	87.36	85.13	86.44 ± 1.16
P16	87.80	88.67	86.57	87.68 ± 1.06
P18	92.48	92.61	90.45	91.85 ± 1.21
P41	87.79	88.16	85.71	87.22 ± 1.32

**Table 12:** Classes with frequent recognition errors on the SLR500 test set.

Class ID	Gloss	Errors	Total Samples	Error Rate (%)
270	Teeth	21	22	95.45
111	Many	20	22	90.91
194	Purple	19	24	79.17
145	Bad	18	25	72.00
267	Nose	18	22	81.82
15	Sign/Mark	17	25	68.00
192	Cyan/Blue-green	17	25	68.00
129	Soft	16	21	76.19
370	Air conditioner	16	25	64.00
410	Button	16	23	69.57

**Table 13:** Persistent high-frequency confusion pairs across three random seeds.

True Class → Predicted Class	Seed 42	Seed 123	Seed 3407	Total
Younger sister → Older sister	11	9	15	35
Object → Short	11	9	13	33
Younger brother → Older brother	10	11	11	32
Cigarette → Mother	11	10	11	32
Thin → Old	8	10	13	31
Civil affairs → Democracy	8	7	15	30
Real → True	9	9	7	25
Son → Daughter	9	8	8	25
Bad → Good	7	8	9	24
Earring → Woman/Girl	9	9	6	24

**Table 14:** Error-rate changes of multimodal fusion configurations during modality-effectiveness evaluation.

Configuration	Top-1 Acc. (%)	Error Rate (%)	ΔError (%)
Skeleton-only	55.3	44.7	–
RGB–Skeleton (simple fusion)	54.0	46.0	+1.3
RGB–Skeleton (MAGF)	54.0	46.0	+1.3
RGB–Skeleton (residual fusion)	53.3	46.7	+2.0
RGB–Skeleton–Depth (MAGF)	2.24	97.76	+53.06

**Table 15:** Sample-level prediction transition statistics between the skeleton-based reference model and the evaluated RGB–Skeleton MAGF fusion model.

Comparison Item	Number of Samples	Percentage (%)
Skeleton reference correct, fusion incorrect	4558	37.48
Skeleton reference incorrect, fusion correct	259	2.13
Both correct	5946	48.88
Both incorrect	1399	11.51

## Data Availability

The dataset used in this study is publicly available. The processed metadata and experimental records generated during the study are available from the corresponding author upon reasonable request, subject to the dataset license and access conditions.

## Abbreviations

The following abbreviations are used in this manuscript:

SLR	Sign Language Recognition
ISLR	Isolated Sign Language Recognition
CSLR	Continuous Sign Language Recognition
RGB	Red–Green–Blue
CTC	Connectionist Temporal Classification
WLASL	Word-Level American Sign Language
MS-ASL	Microsoft American Sign Language
AUTSL	Ankara University Turkish Sign Language
I3D	Inflated 3D ConvNet
3D ResNet	Three-Dimensional Residual Network
TSN	Temporal Segment Networks
ST-GCN	Spatial–Temporal Graph Convolutional Network
SL-GCN	SL-GCN branch from SAM-SLR-v2
SAM-SLR	Skeleton Aware Multi-Modal Sign Language Recognition
MAGF	Modal-Adaptive Gated Fusion
MLP	Multi-Layer Perceptron
TCN	Temporal Convolutional Network
Conv1D	One-Dimensional Convolution
LN	Layer Normalization
ReLU	Rectified Linear Unit
Top-1	Top-1 Accuracy
Top-5	Top-5 Accuracy
MACs	Multiply–Accumulate Operations
GMACs	Giga Multiply–Accumulate Operations
SD	Standard Deviation
CUDA	Compute Unified Device Architecture
GPU	Graphics Processing Unit

## References

[1] Koller O. (2020). Quantitative Survey of the State of the Art in Sign Language Recognition. arXiv.

[2] Bragg D., Koller O., Bellard M., Berke L., Boudreault P., Braffort A., Caselli N., Huenerfauth M., Kacorri H., Verhoef T. Sign Language Recognition, Generation, and Translation: An Interdisciplinary Perspective. Proceedings of the 21st International ACM SIGACCESS Conference on Computers and Accessibility, ASSETS’19.

[3] Camgoz N.C., Koller O., Hadfield S., Bowden R. Sign Language Transformers: Joint End-to-End Sign Language Recognition and Translation. Proceedings of the IEEE/CVF Conference on Computer Vision and Pattern Recognition (CVPR).

[4] Li D., Rodriguez C., Yu X., Li H. Word-Level Deep Sign Language Recognition from Video: A New Large-Scale Dataset and Methods Comparison. Proceedings of the IEEE Winter Conference on Applications of Computer Vision (WACV).

[5] Joze H.R.V., Koller O. (2018). MS-ASL: A Large-Scale Data Set and Benchmark for Understanding American Sign Language. arXiv.

[6] Sincan O.M., Keles H.Y. (2020). AUTSL: A Large Scale Multi-Modal Turkish Sign Language Dataset and Baseline Methods. IEEE Access.

[7] Simonyan K., Zisserman A. Two-Stream Convolutional Networks for Action Recognition in Videos. Proceedings of the Advances in Neural Information Processing Systems (NeurIPS).

[8] Tran D., Bourdev L., Fergus R., Torresani L., Paluri M. Learning Spatiotemporal Features with 3D Convolutional Networks. Proceedings of the IEEE International Conference on Computer Vision (ICCV).

[9] Szegedy C., Vanhoucke V., Ioffe S., Shlens J., Wojna Z. Rethinking the Inception Architecture for Computer Vision. Proceedings of the IEEE Conference on Computer Vision and Pattern Recognition (CVPR).

[10] Carreira J., Zisserman A. Quo Vadis, Action Recognition? A New Model and the Kinetics Dataset. Proceedings of the IEEE Conference on Computer Vision and Pattern Recognition (CVPR).

[11] Hara K., Kataoka H., Satoh Y. Can Spatiotemporal 3D CNNs Retrace the History of 2D CNNs and ImageNet?. Proceedings of the IEEE Conference on Computer Vision and Pattern Recognition (CVPR).

[12] Wang L., Xiong Y., Wang Z., Qiao Y., Lin D., Tang X., Van Gool L. (2016). Temporal Segment Networks: Towards Good Practices for Deep Action Recognition. Computer Vision–ECCV 2016.

[13] Feichtenhofer C., Fan H., Malik J., He K. SlowFast Networks for Video Recognition. Proceedings of the IEEE/CVF International Conference on Computer Vision (ICCV).

[14] Sun C., Myers A., Vondrick C., Murphy K., Schmid C. VideoBERT: A Joint Model for Video and Language Representation Learning. Proceedings of the IEEE/CVF International Conference on Computer Vision (ICCV).

[15] Wang X., Girshick R., Gupta A., He K. Non-Local Neural Networks. Proceedings of the IEEE Conference on Computer Vision and Pattern Recognition (CVPR).

[16] Vaswani A., Shazeer N., Parmar N., Uszkoreit J., Jones L., Gomez A.N., Kaiser L., Polosukhin I. Attention Is All You Need. Proceedings of the Advances in Neural Information Processing Systems (NeurIPS).

[17] Dosovitskiy A., Beyer L., Kolesnikov A., Weissenborn D., Zhai X., Unterthiner T., Dehghani M., Minderer M., Heigold G., Gelly S. An Image Is Worth 16x16 Words: Transformers for Image Recognition at Scale. Proceedings of the International Conference on Learning Representations (ICLR).

[18] Bertasius G., Wang H., Torresani L. Is Space-Time Attention All You Need for Video Understanding?. Proceedings of the International Conference on Machine Learning (ICML).

[19] Defferrard M., Bresson X., Vandergheynst P. Convolutional Neural Networks on Graphs with Fast Localized Spectral Filtering. Proceedings of the Advances in Neural Information Processing Systems (NeurIPS).

[20] Kipf T.N., Welling M. Semi-Supervised Classification with Graph Convolutional Networks. Proceedings of the International Conference on Learning Representations (ICLR).

[21] Veličković P., Cucurull G., Casanova A., Romero A., Liò P., Bengio Y. Graph Attention Networks. Proceedings of the International Conference on Learning Representations (ICLR).

[22] Yan S., Xiong Y., Lin D. Spatial Temporal Graph Convolutional Networks for Skeleton-Based Action Recognition. Proceedings of the AAAI Conference on Artificial Intelligence.

[23] Shi L., Zhang Y., Cheng J., Lu H. Two-Stream Adaptive Graph Convolutional Networks for Skeleton-Based Action Recognition. Proceedings of the IEEE/CVF Conference on Computer Vision and Pattern Recognition (CVPR).

[24] Cheng K., Zhang Y., He X., Chen W., Cheng J., Lu H. Skeleton-Based Action Recognition with Shift Graph Convolutional Network. Proceedings of the IEEE/CVF Conference on Computer Vision and Pattern Recognition (CVPR).

[25] Chen Y., Zhang Z., Yuan C., Li B., Deng Y., Hu W. Channel-Wise Topology Refinement Graph Convolution for Skeleton-Based Action Recognition. Proceedings of the IEEE/CVF International Conference on Computer Vision (ICCV).

[26] Jiang S., Sun B., Wang L., Bai Y., Li K., Fu Y. Skeleton Aware Multi-Modal Sign Language Recognition. Proceedings of the IEEE/CVF Conference on Computer Vision and Pattern Recognition Workshops (CVPRW).

[27] Hu H., Zhao W., Zhou W., Wang Y., Li H. SignBERT: Pre-Training of Hand-Model-Aware Representation for Sign Language Recognition. Proceedings of the IEEE/CVF International Conference on Computer Vision (ICCV).

[28] Plizzari C., Cannici M., Matteucci M. (2021). Skeleton-Based Action Recognition via Spatial and Temporal Transformer Networks. Comput. Vis. Image Underst..

[29] Zhou H., Zhou W., Zhou Y., Li H. (2022). Spatial-Temporal Multi-Cue Network for Sign Language Recognition and Translation. IEEE Trans. Multimed..

[30] Huang J., Zhou W., Zhang Q., Li H., Li W. Video-Based Sign Language Recognition without Temporal Segmentation. Proceedings of the AAAI Conference on Artificial Intelligence.

[31] Pu J., Zhou W., Li H. Iterative Alignment Network for Continuous Sign Language Recognition. Proceedings of the IEEE/CVF Conference on Computer Vision and Pattern Recognition (CVPR).

[32] Pu J., Zhou W., Hu H., Li H. Boosting Continuous Sign Language Recognition via Cross Modality Augmentation. Proceedings of the 28th ACM International Conference on Multimedia (ACM MM).

[33] Baltrušaitis T., Ahuja C., Morency L.P. (2019). Multimodal Machine Learning: A Survey and Taxonomy. IEEE Trans. Pattern Anal. Mach. Intell..

[34] Tsai Y.H.H., Bai S., Liang P.P., Kolter J.Z., Morency L.P., Salakhutdinov R. Multimodal Transformer for Unaligned Multimodal Language Sequences. Proceedings of the 57th Annual Meeting of the Association for Computational Linguistics (ACL).

[35] Xiao Z., Li Z., Zhao Y., Liu Y., Zhang Z., Jia W. (2026). Learning Dual Modality Interactions for Event-Based Motion Deblurring. IEEE Trans. Multimed..

[36] Liu D., Li S., Xiao Z., An P., Shan C. (2025). L3FMamba: Low-Light Light Field Image Enhancement with Prior-Injected State Space Models. IEEE Signal Process. Lett..

[37] Rosenstein M.T., Marx Z., Kaelbling L.P., Dietterich T.G. To Transfer or Not to Transfer. Proceedings of the NIPS Workshop on Transfer Learning.

[38] Ruder S. (2017). An Overview of Multi-Task Learning in Deep Neural Networks. arXiv.

[39] He K., Zhang X., Ren S., Sun J. Deep Residual Learning for Image Recognition. Proceedings of the IEEE Conference on Computer Vision and Pattern Recognition (CVPR).

[40] Ioffe S., Szegedy C. Batch Normalization: Accelerating Deep Network Training by Reducing Internal Covariate Shift. Proceedings of the International Conference on Machine Learning (ICML).

[41] Srivastava N., Hinton G., Krizhevsky A., Sutskever I., Salakhutdinov R. (2014). Dropout: A Simple Way to Prevent Neural Networks from Overfitting. J. Mach. Learn. Res..

